# Japanese Encephalitis Virus NS4A Protein Interacts with PTEN-Induced Kinase 1 (PINK1) and Promotes Mitophagy in Infected Cells

**DOI:** 10.1128/spectrum.00830-22

**Published:** 2022-05-23

**Authors:** Anshu Agarwal, Mohd. Faraz Alam, Brohmomoy Basu, Sabyasachi Pattanayak, Shailendra Asthana, Gulam Hussain Syed, Manjula Kalia, Sudhanshu Vrati

**Affiliations:** a Translational Health Science and Technology Institute, Faridabad, India; b Institute of Life Sciences, Bhubaneswar, India; c Regional Centre for Biotechnology, Faridabad, India; Thomas Jefferson University

**Keywords:** yeast-two-hybrid, protein interactome, flavivirus, mitochondria

## Abstract

The nonstructural protein 4A (NS4A) of flaviviruses has been implicated as a “central organizer” of the membrane-bound replication complex during virus replication. However, its role in the host responses to virus infection is not understood. Using the yeast-two-hybrid library screen, we identified a multitude of host proteins interacting with the Japanese encephalitis virus (JEV) NS4A protein. Several of these interacting proteins are known to localize to the mitochondria. One of these proteins was PTEN-induced kinase 1 (PINK1), a serine/threonine-protein kinase known for its role in mitophagy. Here, we demonstrate the JEV-NS4A localization to the mitochondria and its interaction with PINK1 in Huh7 cells during JEV infection. The JEV-infected cells showed an enhanced mitophagy flux with a concomitant decline in the mitochondrial mass. We present data showing that JEV-NS4A alone was sufficient to induce mitophagy. Interference with mitochondrial fragmentation and mitophagy resulted in reduced virus propagation. Overall, our study provides the first evidence of mitochondrial quality control dysregulation during JEV infection, largely mediated by its NS4A protein.

**IMPORTANCE** The JEV-infected mammalian cells show an enhanced mitophagy flux with a concomitant decline in the mitochondrial mass. We show that the NS4A protein of JEV localized to the mitochondria and interacted with PINK1 in Huh7 cells during infection with the virus and demonstrate that JEV-NS4A alone is sufficient to induce mitophagy. The study provides the first evidence of mitochondrial quality control dysregulation during JEV infection, largely mediated by its NS4A protein.

## INTRODUCTION

Japanese encephalitis virus (JEV) is the leading cause of viral encephalitis in Asia, where a population of around 3 billion people is at risk of infection with the virus (1). Worldwide, ~70,000 Japanese encephalitis (JE) cases are reported each year, resulting in up to 20,000 deaths (2). The disease is characterized by fever, headache, and malaise as the nonspecific symptoms, while neck rigidity, cachexia, convulsions, and paralysis are specific symptoms frequently leading to a fatal outcome. There is presently no specific therapy for the disease, and treatment is focused on relieving severe clinical signs and providing support for overcoming the infection (3). Available vaccines have had limited success in preventing the disease (4, 5). It is important to understand the biology of virus replication and the cellular mechanisms of viral pathogenesis to develop novel anti-JEV therapeutics.

JEV is an enveloped virus belonging to the *Flaviviridae* family, which also includes medically important viruses like dengue, yellow fever, West Nile, hepatitis C (HCV), and zika viruses (6). The virus has a positive-sense, ~11-kb single-stranded RNA genome, which is translated into a polyprotein precursor cleaved subsequently by the host and viral proteases into three structural (envelope [E], pre-membrane [prM], and capsid [C]) and seven nonstructural proteins (NS1, NS2A, NS2B, NS3, NS4A, NS4B, and NS5). While the structural proteins form the mature virion particle, the nonstructural proteins are involved in viral genome replication, virus assembly, egress, and evasion of host immune responses (7). NS1 is produced in a secretory form and is a part of the viral replication complex; NS3 acts as both a viral protease (with required cofactor NS2B) and a viral ATP-dependent helicase, whereas NS5 has the RNA-dependent RNA polymerase (RdRp) and methyltransferase activities. NS2A, NS2B, NS4A, and NS4B are small hydrophobic proteins and are the least characterized ones (8).

The flavivirus NS4A protein has a vital role in virus replication. Dengue virus (DENV) NS4A is a highly hydrophobic, 16-kDa transmembrane protein that localizes with the viral replication complex and has been found to induce host membrane alterations resembling the virus-induced membrane rearrangements (9). Interaction between DENV NS4A and cellular vimentin intermediate filaments has been shown to promote replication complex (RC) formation (10). These RCs are located within a virus-induced and ER-derived complex membrane network consisting of interconnected lipid vesicles and convoluted membranes (11). The DENV NS4A can alone induce autophagy to prevent cell death and facilitate viral replication (12). To understand the role of the JEV-NS4A protein in virus replication and host cell metabolism, we generated its protein interactome using the yeast-2-hybrid (Y2H) screen. We created the JEV-NS4A interaction network composed of 150 interactions from 75 identified proteins showing a significant involvement of mitochondrial proteins.

Virus infection can impair mitochondrial function and dynamics, either directly through viral proteins or indirectly due to the physiological stresses associated with infection. Many viruses have been shown to target mitochondria, which play a significant role in cellular antiviral signaling, and disruption of cellular antiviral signaling can facilitate the establishment of a cellular environment conducive for viral propagation. To prevent the spread of the virus infection, the host defense may promote the death of the infected cell by triggering apoptosis, autophagy, or necroptosis. Viruses have been shown to modulate (block/promote) these processes for persistence and evading the host immunological response (13). PTEN-induced kinase 1 (PINK1), one of the proteins identified in our JEV-NS4A interactome, has been implicated in the activation of mitophagy, the mitochondria-selective autophagy process required to maintain cellular homeostasis (14). Mitophagy-mediated clearance of damaged and dysfunctional mitochondria is essential to protect cellular viability by inhibiting the release of proapoptotic proteins (15). Therefore, we carried out a detailed characterization of JEV-NS4A interaction with PINK1 to explore its role in the JEV life cycle and disease pathogenesis. In this study, we show that the JEV-NS4A localizes to the mitochondria and interacts with PINK1. The virus infection led to the downregulation of PINK1 expression and enhanced the mitophagy flux with a concomitant decline in the mitochondrial mass. Interfering with the JEV-induced mitochondrial fragmentation and mitophagy resulted in significantly reduced virus propagation. Our results show that the JEV nonstructural protein NS4A has a role in the mitochondrial dynamics during the virus’s infection of mammalian cells.

## RESULTS

### The mouse JEV-NS4A interactome mapping.

**FIG 1:**
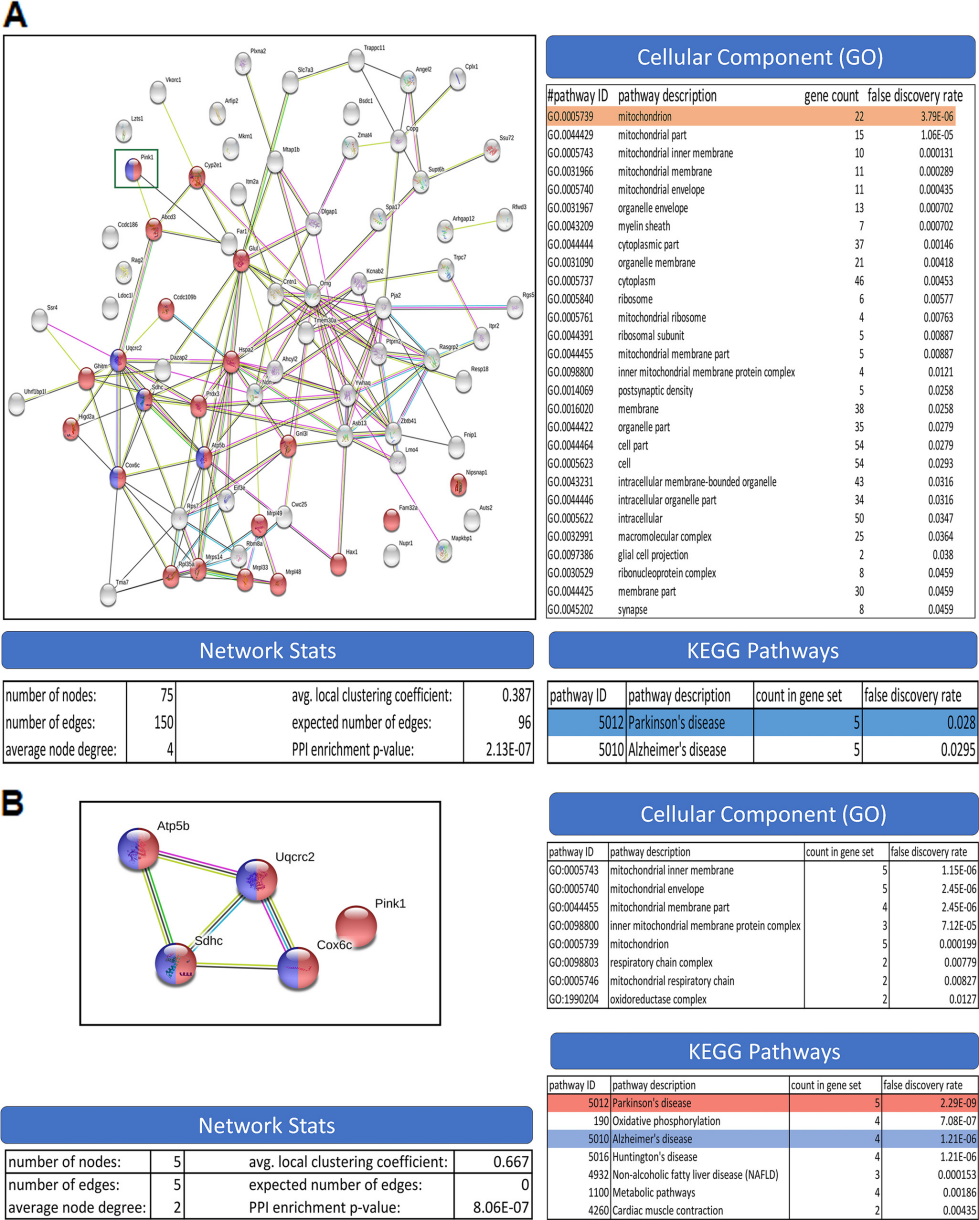
The mouse JEV-NS4A interactome. (A) The Y2H screen of the mouse brain cDNA library with JEV-NS4A identified 75 interacting protein partners. Each node in the left panel represents an identified protein, and edges represent protein-protein associations not necessarily representing physical interactions. The pathway enrichment analysis (right panel) was conducted with the help of the STRING (version 10.5) bioinformatics tool at the confidence level of 0.15. A *P* value of <0.05 was used as the cutoff criterion. The red nodes in the interaction map (left panel) represent proteins from the mitochondrion cellular component, and the purple nodes denote the proteins enriched in Parkinson’s disease. (B) The subinteractome of the genes enriched for the Parkinson’s disease (PD) pathway (left panel). The pathway enrichment analysis (right panel) was conducted with the help of the STRING (version 10.5) bioinformatics tool at the confidence level of 0.15. A *P* value of <0.05 was used as the cutoff criterion. The red nodes (left panel) depict the proteins enriched in PD, while the purple nodes depict proteins enriched in Alzheimer’s disease.

The Y2H screening was carried out to identify the cellular proteins that may interact with JEV-NS4A. The JEV-NS4A cDNA was cloned into the bait vector and used against the mouse brain cDNA library as the prey. A total of 120 colonies so obtained were streaked on stringent selective plates, and the colonies growing after 5 days were replica plated for the X-Gal assay (see Fig. S1 in the supplemental material). Ninety-nine such clones were sequenced, of which 75 were identified as containing the mouse cDNA inserts. The identified genes (Table S1) from the cDNA inserts were used to generate an interactome map (Fig. 1A). These were subsequently enriched for the various Gene Ontology (GO) categories. Although there were no significant pathway enrichments observed in the biological process and the molecular function categories in GO, ~30% of annotated genes (22 out of 75) were enriched for the cellular component (GO: 0005739) of mitochondria. The genes were also enriched for two KEGG (Kyoto Encyclopedia of Genes and Genomes) pathways of Parkinson’s (5 genes) as well as Alzheimer’s (5 genes) diseases. The five genes enriched for Parkinson’s disease (PD) were further annotated, and it was seen that except for PINK1 (our gene of interest), which was unique to PD, the remaining four genes were common to other neurological conditions like Huntington’s disease, Alzheimer’s disease, oxidative phosphorylation, and metabolic pathways (Fig. 1B).

### Subcellular localization of JEV-NS4A.

**FIG 2:**
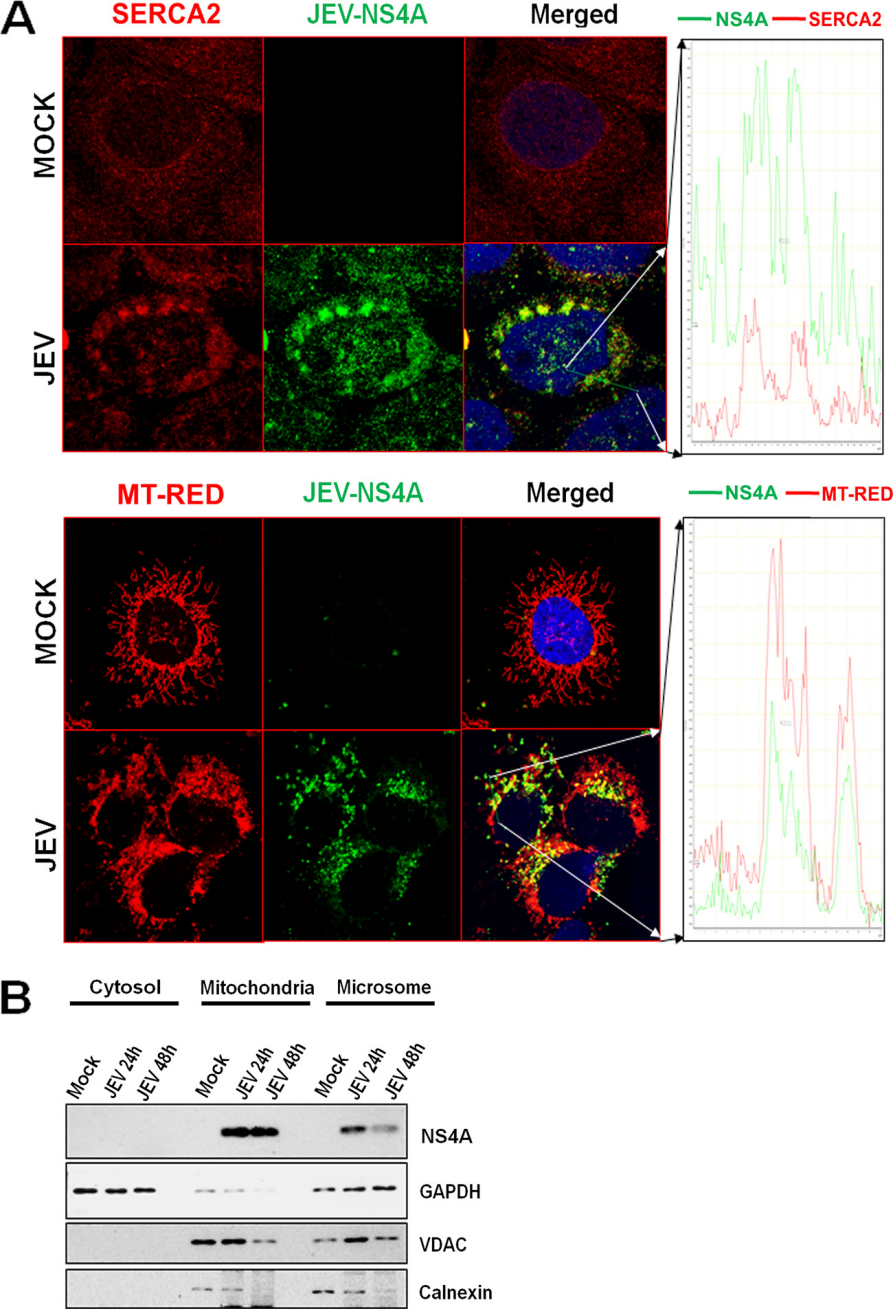
Subcellular localization of JEV-NS4A to ER and mitochondria. (A) Huh7 cells were infected with JEV (MOI 1) and 48 h later stained with MitoTracker Red, fixed with 4% PFA, and immune-stained with JEV-NS4A and SERCA2 antibody (ER marker). These cells were then stained with respective Alexa fluor secondary antibodies, stained with DAPI for nuclei, and visualized under a confocal microscope (left panel). The colocalization is depicted as a line profile in the right panel. (B) Mock- and JEV-infected Huh7 cells harvested at 24 and 48 hpi were subjected to fractionation. We performed Western blotting on the cytosolic, mitochondrial, and microsomal fractions with JEV-NS4A antibody. GAPDH, VDAC, and calnexin were used as markers for cytosol, mitochondria, and ER, respectively.

NS4A is a membrane-associated protein known to serve as a scaffold for the formation of the replication complexes of flaviviruses (8). We investigated the localization of JEV-NS4A to the endoplasmic reticulum (ER) and mitochondria. We established the localization of NS4A to the ER by demonstrating its colocalization with sarco(endo)plasmic reticulum calcium ATPase2 (SERCA2), which is localized primarily at the ER (Pearson’s coefficient of 0.75) (Fig. 2A). To determine if NS4A localizes to the mitochondria, we examined its colocalization with MitoTracker Red-labeled mitochondria. We observed that the JEV-NS4A localized to the mitochondria, as well (Fig. 2A). To further validate these findings, we isolated subcellular fractions from the mock- and JEV-infected Huh7 cells. Western blotting showed the presence of JEV-NS4A in the ER and mitochondrial fractions, which was consistent with our colocalization data (Fig. 2B).

### JEV-NS4A and PINK1 interaction.

**FIG 3:**
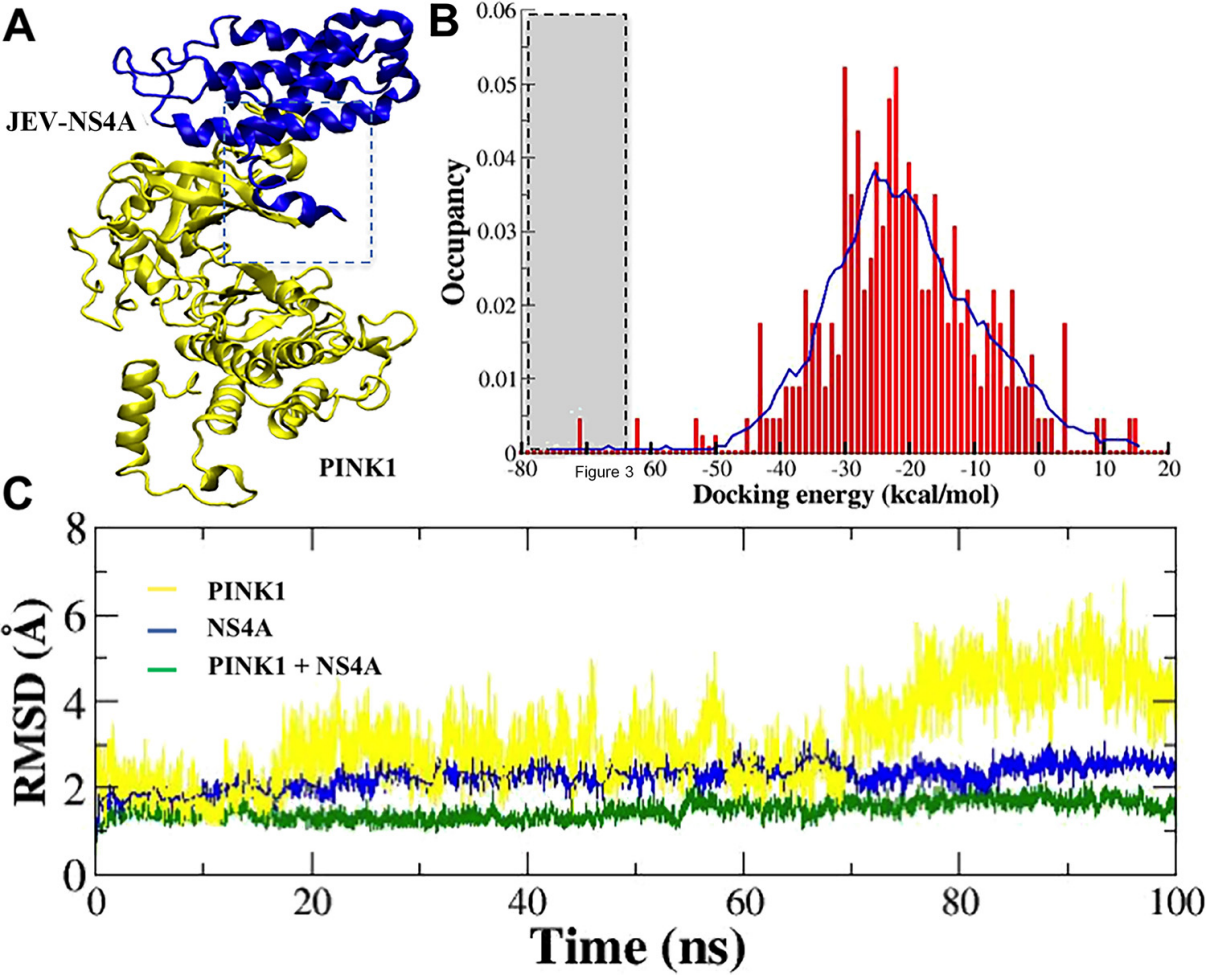
Interaction of JEV-NS4A with PINK1 *in silico*. The molecular docking studies were undertaken followed by molecular dynamic simulations to predict the NS4A-PINK1 complex stability. (A) The best-docked pose in the form of the lowest docking energy is shown in the cartoon. The JEV NS4A is shown in blue, whereas PINK1 is shown in yellow. The dotted rectangle denotes the interaction zone in the complex. (B) Histogram of protein-protein docking score. The dotted rectangular box is the region selected for NS4A-PINK1 complex analysis. (C) The molecular dynamic simulation graph highlighting the stability of the NS4A-PINK1 trajectory is shown in green. PINK1 alone is shown in yellow, and JEV-NS4A alone is shown in blue.

*In silico* studies involving molecular docking were undertaken to examine the plausibility of JEV-NS4A interaction with PINK1 as seen in the Y2H screen. As no crystal structure of JEV-NS4A was available, template was taken from I-TASSER. For the human PINK1, the previously described structure (PDB-ID 6EQI) was used. Using the different docking algorithms and the various parameters described in Materials and Methods, the most likely poses of the PINK1-NS4A complex were generated and the best-docked pose was identified (Fig. 3A) based on docked energy among all the poses (Fig. 3B). The best pose having −70 to −80 kcal/mol docking energy was used for molecular dynamic simulations (100 ns) (Fig. 3C). Both the protein structures displayed an initial rearrangement that was followed by convergence to a root mean square deviation (RMSD) plateau, showing close relation between the two structures, indicative of protein-protein interaction with high docking energy of approximately −75.0 kcal/mol.

**FIG 4:**
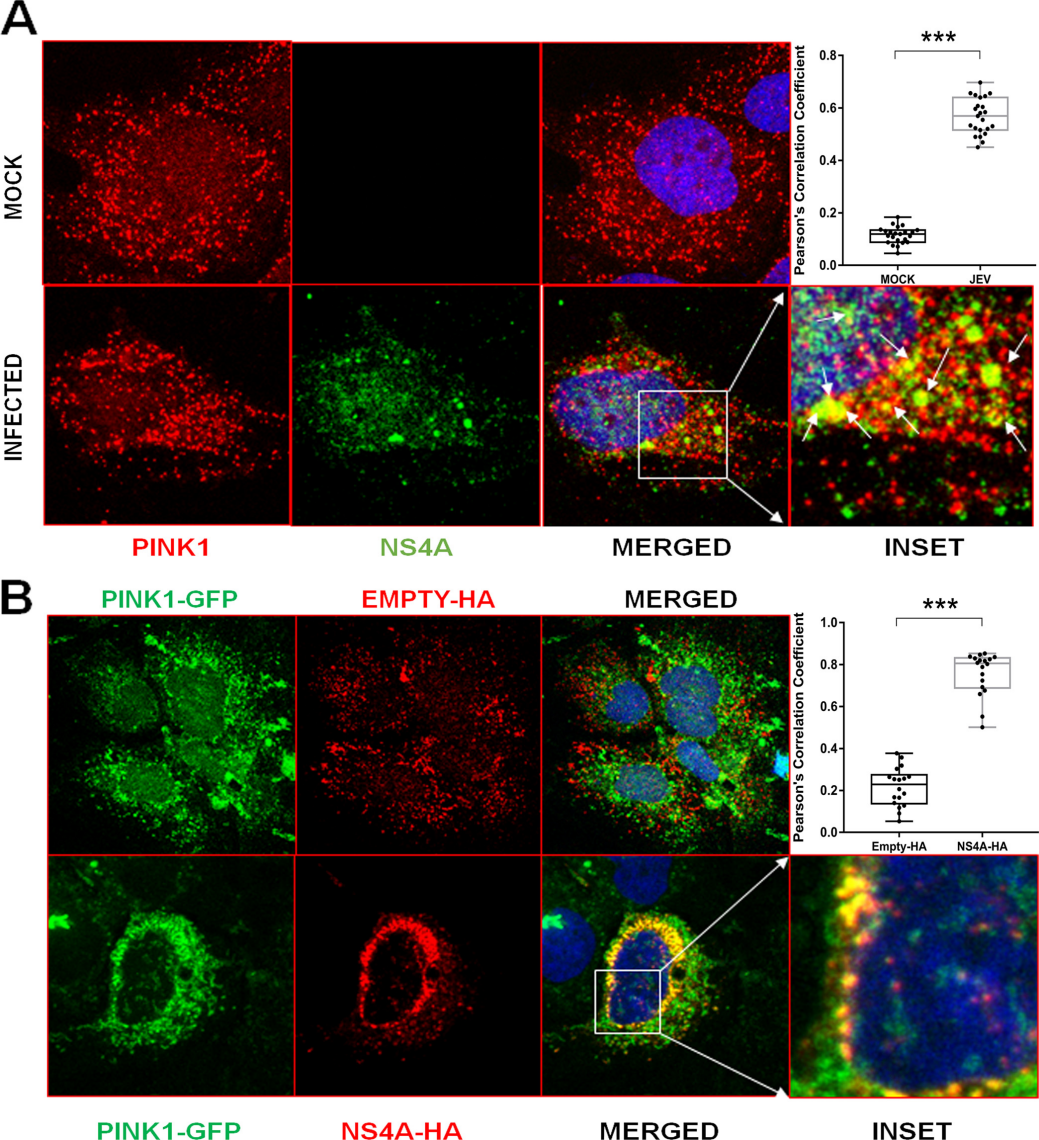
Colocalization of JEV-NS4A with PINK1. (A) Huh7 cells were mock-infected or infected with JEV (MOI 1) and fixed at 48 hpi followed by immunostaining with antibodies against PINK1, JEV-NS4A, and respective Alexa fluor secondary antibodies. Images were taken using the Leica TCS SP8 confocal microscope. The inset has zoomed-in yellow spots depicting JEV-NS4A and PINK1 colocalization marked by the arrows. The quantitation of colocalization by Pearson’s correlation coefficient is depicted as a graph in the top right corner. A total of 21 region of interests (ROIs) each (1 cell equivalent to 1 ROI) were quantified. The data between the mock- and JEV-infected cells were compared by the Student’s *t* test (***, *P* < 0.001). (B) Huh7 cells were transfected with plasmids expressing JEV-NS4A tagged with HA (NS4A-HA) or the empty-HA vector and GFP-tagged PINK1 (PINK1-GFP). The cells were fixed 48 h posttransfection and immunostained with anti-HA antibody followed by the corresponding Alexa fluor secondary antibody. Images were captured using the Leica TCS SP8 confocal microscope. The quantitation of colocalization by Pearson’s correlation coefficient is depicted as a graph in the top right corner. A total of 18 ROIs each (1 cell equivalent to 1 ROI) were quantified. The data between the empty-HA and NS4-HA transfected cells were compared with the Student’s *t* test (***, *P* < 0.001).

Confocal microscopy-based colocalization studies on JEV-infected cells were undertaken to validate the *in silico* findings indicating the high probability of the JEV-NS4A interaction with PINK1. Huh7 cells were infected with JEV and stained for the JEV-NS4A and PINK1 proteins. Confocal microscopy showed a significant colocalization between PINK1 and NS4A (Pearson’s coefficient of 0.6) in JEV-infected cells (Fig. 4A). To substantiate this further, Huh7 cells were transfected with the plasmids expressing hemagglutinin (HA)-tagged JEV-NS4A (NS4A-HA) and green fluorescent protein (GFP)-tagged PINK1. These cells were stained with antibodies to HA tag and visualized under a confocal microscope (Fig. 4B). The exogenously expressed JEV-NS4A and PINK1 proteins showed a significant colocalization (Pearson’s coefficient of 0.65).

**FIG 5:**
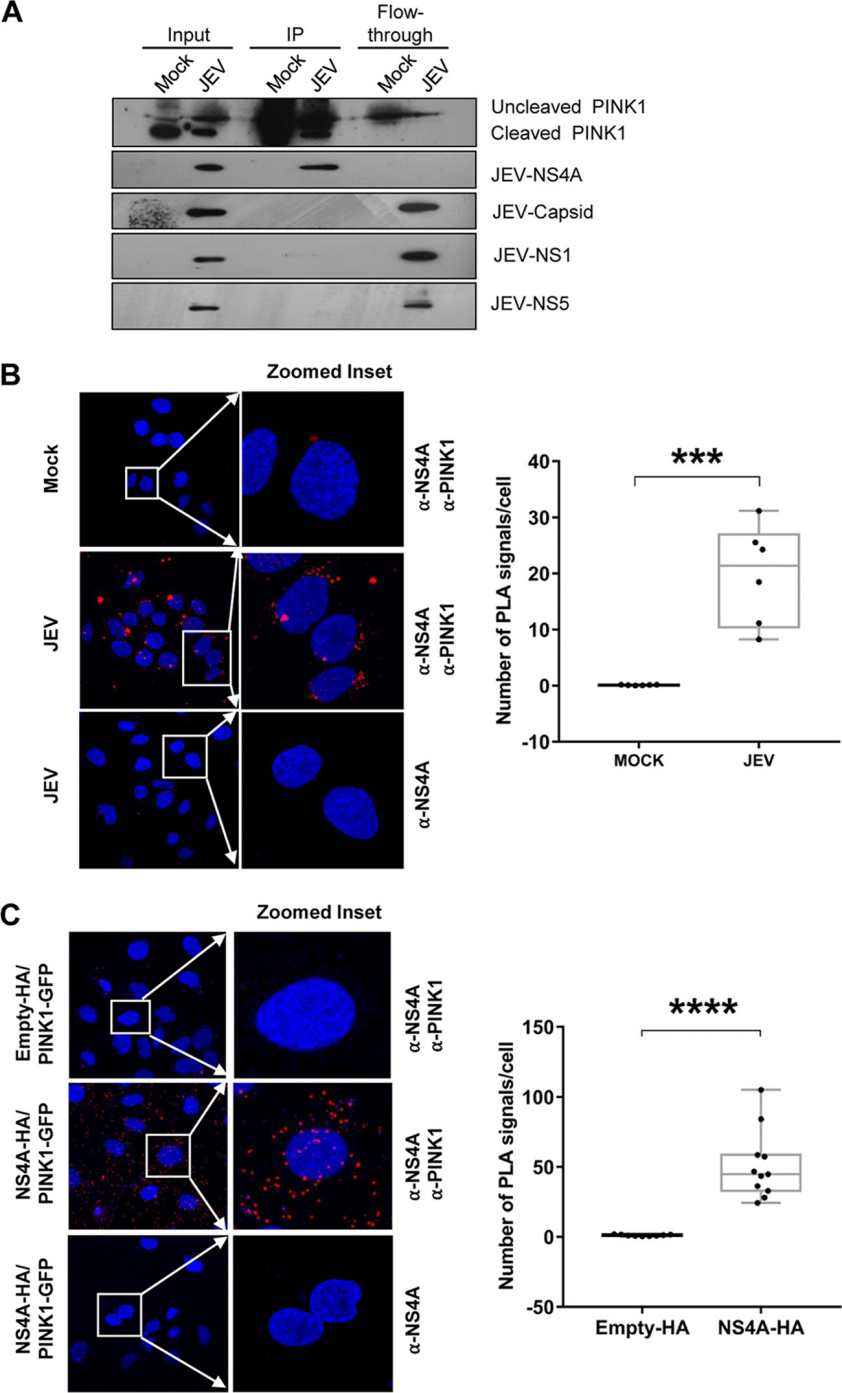
Interaction of JEV-NS4A with PINK1 in mammalian cells. (A) HEK293T cells were transfected with the plasmid expressing GFP-tagged PINK1 and 24 h later were mock-infected or infected with JEV (MOI 3). Cell lysates were prepared 48 hpi and used for pulldown with GFP-trap beads. The immunoprecipitate (IP), flowthrough, and input (10% of lysate used for immunoprecipitation) were subjected to Western blotting. (B) Huh7 cells were mock-infected or infected with JEV (MOI 3) and fixed at 24 hpi. These cells were subjected to the Duolink proximity ligation assay (PLA). The cell nucleus was stained with DAPI. Imaging was done using the Leica TCS SP8 confocal microscope. Positive interaction between NS4A and PINK1 is detected as discrete fluorescent red spots (PLA signals). Representative confocal images are shown in the left panel. The right panel depicts the quantification of the number of PLA signals per cell in six different fields. Image analysis was done using ImageJ software. (C) Huh7 cells were cotransfected with plasmids expressing PINK1-GFP and NS4A-HA or empty-HA, fixed at 48 hpi, and subjected to PLA. Imaging was done using the Leica TCS SP8 confocal microscope. Representative confocal images are shown in the left panel. The right panel depicts the quantification of the number of PLA signals per cell in nine different fields. The Student’s *t* test was used to determine the statistical significance of the difference between the JEV-infected and mock-infected cells (***, *P* < 0.001).

To further validate the JEV-NS4A and PINK1 interaction in mammalian cells, immunoprecipitation was performed with lysates obtained from HEK293T cells transfected with the plasmid expressing PINK1 fused to GFP followed by JEV infection (Fig. 5A). Immunoprecipitation of PIKN1-GFP resulted in a specific pulldown of only JEV-NS4A, and no other JEV proteins like NS1, NS5, and capsid were immunoprecipitated (Fig. 5A). However, our attempts to pull down endogenous PINK1 in JEV-infected cells using JEV-NS4A antibody, or vice-versa, were not successful.

We used the *in situ* proximity ligation assay (PLA) to demonstrate the interaction of the endogenous PINK1 with JEV-NS4A in the virus-infected cell. PLA is a powerful method capable of detecting the interactions among proteins with higher specificity and sensitivity than coimmunoprecipitation. While the mock-infected Huh7 cells displayed a very low number of fluorescent dots per cell (average of 0.12), the JEV-infected Huh7 cells showed a significantly high number of fluorescent dots per cell (average of ~20) (Fig. 5B), clearly demonstrating an interaction between the JEV-NS4A and the endogenous PINK1. The fluorescent dots were clustered in the perinuclear region, as previously observed in the colocalization experiments. The PLA on Huh7 cells overexpressing NS4A-HA along with PINK1-GFP further validated the above finding (Fig. 5C). No dots were seen in virus-infected or transfected cells incubated with JEV-NS4A antibody alone.

### JEV infection modulates PINK1 expression.

**FIG 6:**
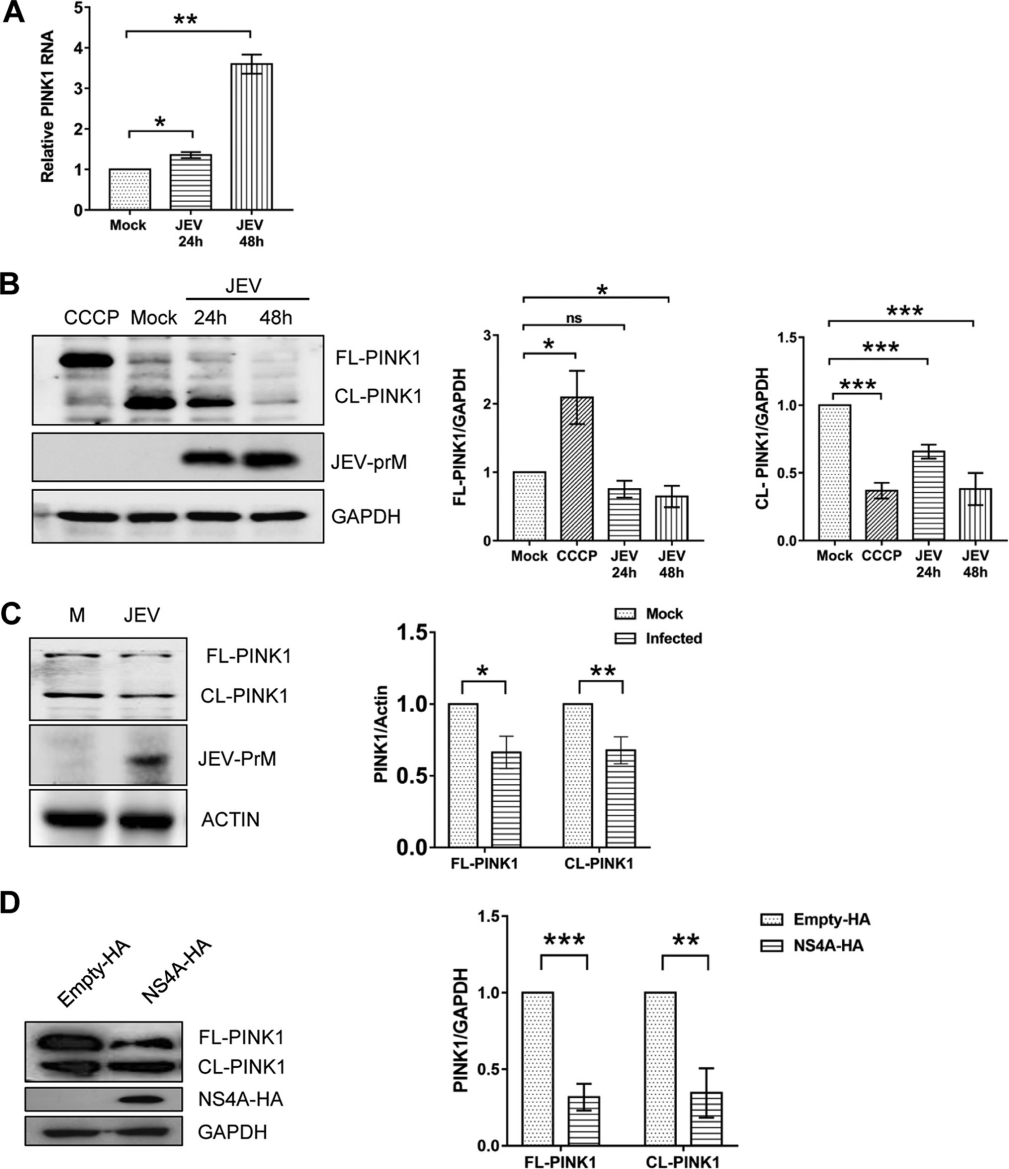
JEV infection modulates PINK1 expression. Huh7 cells were mock-infected or infected with JEV (MOI 1), and the cells were harvested at 24 and 48 hpi. (A) Total RNA isolated from the cells was used to measure the PINK1 mRNA by qRT-PCR. GAPDH was used as the internal control. Levels of PINK1 mRNA in JEV-infected cells relative to those in the mock-infected cells are depicted. (B) The cell lysates were subjected to Western blotting to detect the full-length (FL) and cleaved (CL) forms of PINK1 (left panel). GAPDH was used as the loading control, and JEV-prM was used as the infection marker. CCCP-treated Huh7 cell lysates were used as a positive control for the full-length (FL) PINK1. The right panels show the densitometric analysis of full-length and cleaved PINK1 band intensities. (C) Three-week-old BALB/c mice (*n* = 4) were mock- or JEV-infected by the intraperitoneal mode of injection. The liver tissue was harvested 5 days later and prepared as lysate. The left panel shows the Western blotting of FL- and CL-PINK1 in JEV-infected mouse liver lysate. The JEV-prM was used as the infection marker, and actin was used as the internal loading control. The right panel shows quantification of FL- and CL-PINK1 band intensities of mice tissue by the Western blots. (D) HEK293T cells were transfected with plasmids expressing JEV-NS4A with HA tag (NS4A-HA) or the empty vector expressing only the HA tag (empty-HA). The cell lysates were prepared 48 h later and subjected to Western blotting (left panel). GAPDH was used as the loading control. The right panel shows the quantification of full-length and cleaved PINK1 band intensities of the Western blots. The Western blot data are representative of the three independent experiments whose data are plotted as bar graphs. Statistical analysis was performed using the Student’s *t* test (*, *P* ≤ 0.05; **, *P* ≤ 0.01; ***, *P* ≤ 0.001; ns, not significant).

In a healthy cell, PINK1 protein is translocated to mitochondria, where it is rapidly cleaved by the mitochondrial intramembrane proteases, and the cleaved fragment is exported back to the cytosol to be further degraded by the proteasome, thus maintaining low steady-state levels of PINK1 (16). We studied the expression of PINK1 during JEV infection. A significant increase in PINK1 mRNA levels was seen in the JEV-infected Huh7 cells (Fig. 6A). However, Western blotting showed a downregulation in the levels of full-length PINK1 (FL-PINK1) and cleaved-PINK1 (CL-PINK1) protein during the JEV infection (Fig. 6B). In this experiment, cells treated with the mitochondrial decoupler carbonyl cyanide chlorophenylhydrazone (CCCP) displayed mostly full-length PINK1 as expected. The PINK1 expression profile was also checked in JEV-infected mice tissues. There was a significant decrease in the full-length and cleaved PINK1 protein expression in the liver lysates of virus-infected mice compared to that of the mock-infected control mice (Fig. 6C). Similar results were seen in the mouse brain tissue (Fig. S3).

Next, we examined if JEV-NS4A alone could modulate the PINK1 levels in the cell. For this, HEK293T cells were transfected with the NS4A-HA or the empty-HA expression plasmids. Western blots of the cell lysates showed a significant downregulation of PINK1 in the JEV-NS4A-transfected cells compared to that of the empty vector (empty-HA)-transfected cells (Fig. 6D), indicating that JEV-NS4A alone could downregulate the expression of PINK1 in mammalian cells.

### JEV infection causes mitochondrial fragmentation and a decrease in the mitochondrial mass.

**FIG 7:**
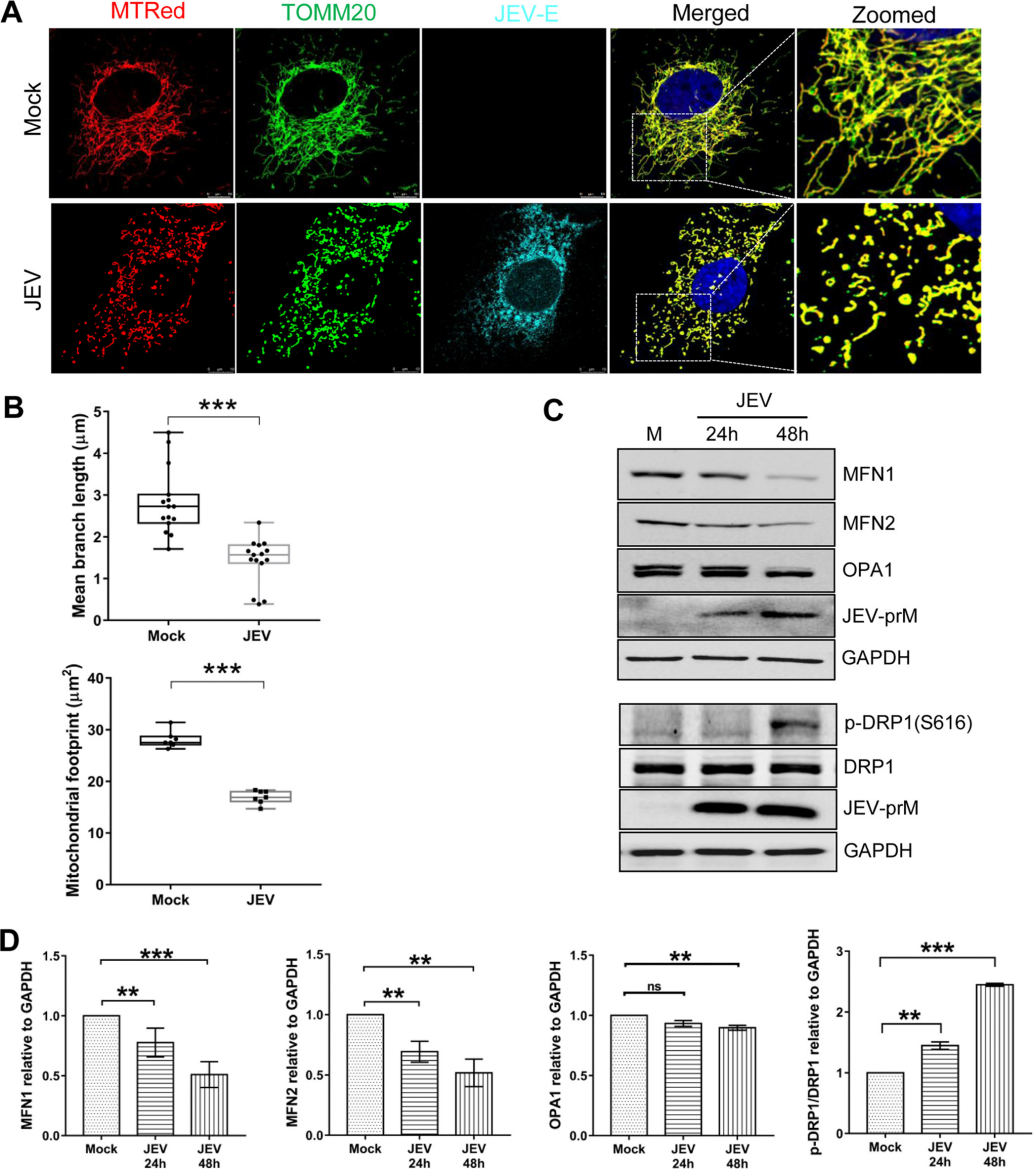
Mitochondrial dynamics in JEV-infected Huh7 cells. Huh7 cells were mock-infected or infected with JEV (MOI 1) and 48 h later stained with the MitoTracker dye before fixation with 4% PFA. Cells were immunostained for TOMM20 and JEV-E proteins. (A) Images taken using the Leica TCS SP8 confocal microscope are shown. The MitoTracker and TOMM20 images were merged to observe the mitochondria shape. (B) Quantification of the mitochondrial branch length and footprint in mock- and JEV-infected cells. (C) Cell lysates obtained from mock- and JEV-infected cells at 24 and 48 hpi were subjected to Western blotting for the mitochondrial fission and fusion proteins as indicated. (D) Quantification of MFN1, MFN2, OPA1, and DRP1 by band intensities, relative to GAPDH, in mock- and JEV-infected cells. The Western blots (in panel C) are representative of the three independent experiments whose data are plotted as bar graphs. Statistical analysis was performed using the Student’s *t* test (*, *P* ≤ 0.05; **, *P* ≤ 0.01; ***, *P* ≤ 0.001; ns, not significant).

The data above showed that JEV-NS4A and PINK1 interacted with each other and were associated with mitochondria. Also, in the JEV-infected cells, the mitochondria distribution was starkly different from that in the mock-infected cells. We, therefore, further studied the mitochondrial morphology in the JEV-infected cells. Costaining of mitochondria with MitoTracker Red and TOMM20 antibody showed a typical tubular mitochondrial network in the mock-infected Huh7 cells. However, JEV-infected cells showed fragmented mitochondria (Fig. 7A). Quantitative analysis confirmed an overall decline in the average mitochondrial length and footprint, indicative of mitochondrial fragmentation and reduction in the mitochondrial mass in the JEV-infected cells (Fig. 7B). The virus-infected cells showed a small yet significant decrease in the expression levels of the mitochondrial outer membrane fusion proteins MFN1 and MFN2 and inner membrane fusion protein OPA1 at 48 h postinfection (hpi). Consistent with this, a significant increase was seen in the Ser616-phosphorylated form of the mitochondrial fission protein DRP1, although we did not observe any change in the total DRP1 levels (Fig. 7C and D). Overall, these data suggested that JEV infection shifted the balance of mitochondrial dynamics towards enhanced fission and promoted DRP1-mediated mitochondrial fragmentation.

### JEV infection induces mitophagy.

**FIG 8:**
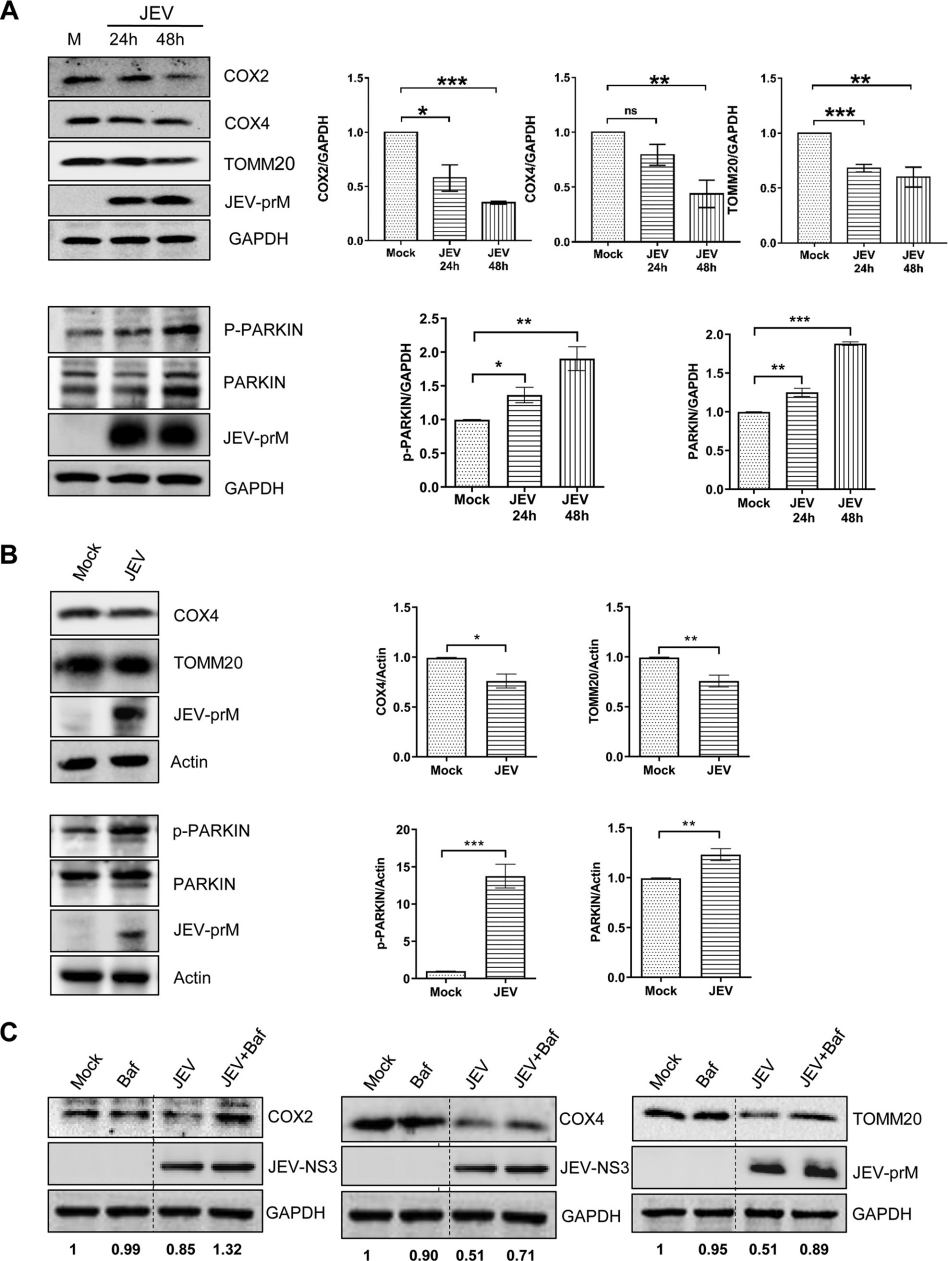
Mitochondrial and mitophagy-related proteins during JEV infection. (A) Huh7 cells were mock-infected or infected with JEV (MOI 1), and cells were harvested at 24 and 48 hpi. The cell lysates were subjected to Western blotting for the indicated proteins. GAPDH was used as the loading control, and JEV-prM was used as the infection marker. The Western blots are representative of the three independent experiments whose data are plotted as bar graphs. (B) Three-weeks-old BALB/c mice (*n* = 4) were infected with JEV or mock-infected by the intraperitoneal mode of injection. The liver tissues were harvested 5 days later and prepared as lysates. The left panels show the Western blotting of indicated proteins in JEV-infected mouse liver lysate. The JEV-prM was used as the infection marker, and actin was used as the internal loading control. The right panel shows quantification of protein band intensities of mouse tissue from the Western blots. (C) Mock- and JEV-infected (MOI 1) Huh7 cells were treated at 30 hpi with bafilomycin for 8 h. The cell lysates were subjected to Western blotting for the indicated proteins. GAPDH was used as the loading control, and JEV-prM or JEV-NS3 was used as the infection marker. The band intensities of COX2, COX4, and TOMM20 compared to GAPDH are shown at the bottom. Statistical analysis was performed using the Student’s *t* test (*, *P* ≤ 0.05; **, *P* ≤ 0.01; ***, *P* ≤ 0.001; ns, not significant).

Mitochondrial fragmentation is a prerequisite of mitophagy where the fragmented mitochondria serve as the good substrate (17). To determine the effect of JEV infection on mitophagy, we studied the expression of mitochondrial proteins COX4 and TOMM20, encoded by the nuclear genome, and COX2, encoded by the mitochondrial genome. Immunoblot analysis revealed that their levels were significantly lower in JEV-infected Huh7 cells (Fig. 8A), suggesting an overall decline in the mitochondrial mass in the virus-infected cells. However, PARKIN, an E3 ubiquitin ligase and a well-known effector of PINK1-mediated mitophagy, was upregulated in the JEV-infected cells (Fig. 8A, lower panel). We also observed high levels of the Ser65 phosphorylated form of PARKIN, which represents active PARKIN phosphorylated by PINK1 (Fig. 8A, lower panel) (18). Bafilomycin treatment of JEV-infected Huh7 cells led to the accumulation of both PINK1 and PARKIN (Fig. S2), further establishing their role in the JEV-triggered mitophagy. Similar observations were made on the expression levels of these proteins in the JEV-infected mice tissue (Fig. 8B). The fact that the downregulation of the mitochondrial proteins was a consequence of mitophagy *per se* and not due to any other factor was confirmed by treatment of the JEV-infected Huh7 cells with bafilomycin (autophagosome-lysosome fusion inhibitor). The bafilomycin treatment resulted in the rescue of the levels of the mitochondrial proteins COX2, COX4, and TOMM20, suggesting that the observed decline in mitochondrial mass is mediated through mitophagy in the JEV-infected cells (Fig. 8C).

**FIG 9:**
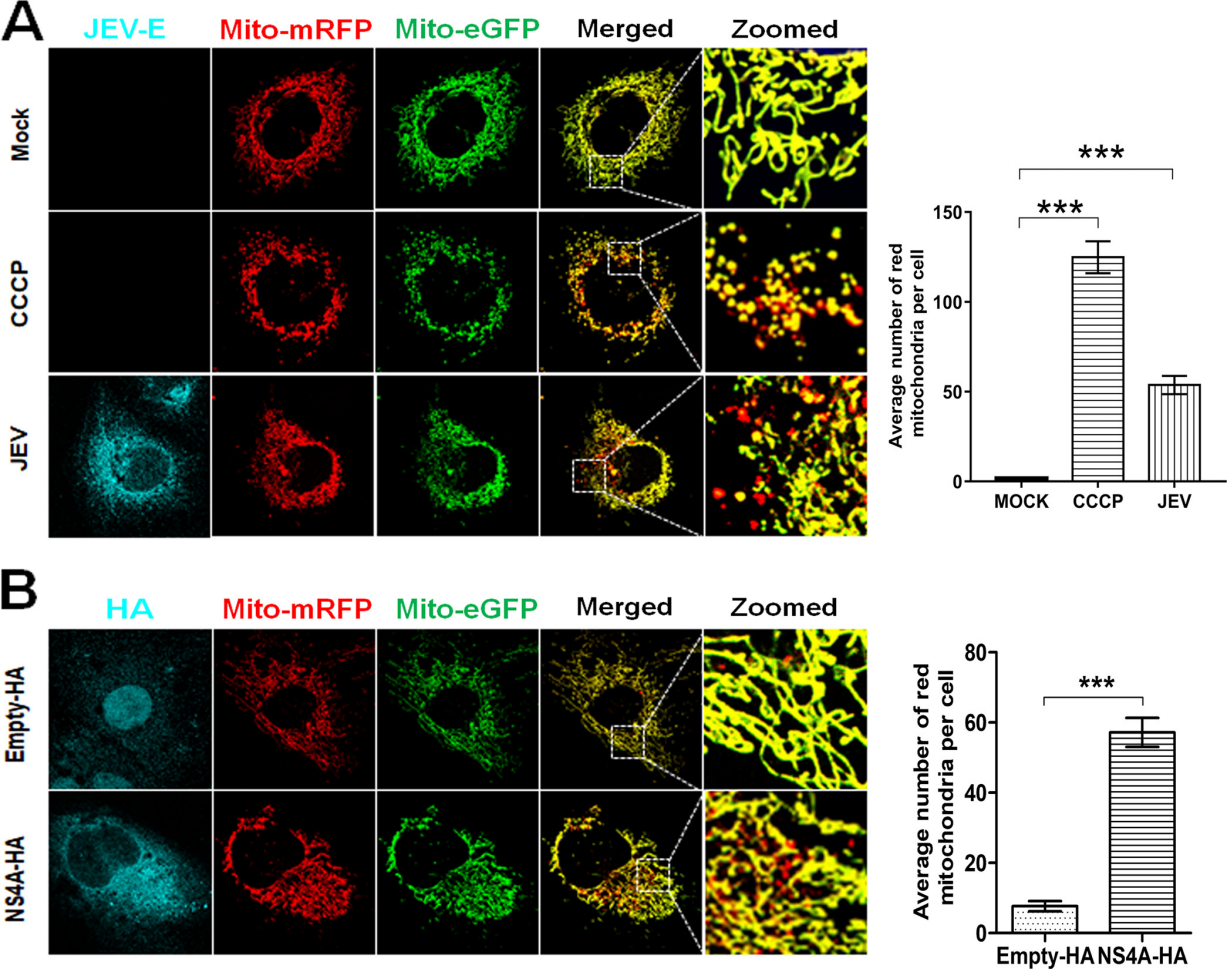
Mitophagy flux in JEV-infected Huh7 cells. (A) Huh7 cells were transfected with the mitophagy reporter plasmid mito-mRFP-EGFP and 24 h later infected with JEV (MOI 3). Huh7 cells treated with CCCP were used as a positive control for mitophagy. The cells were fixed 48 hpi and immunostained with the JEV-E antibody. Images were taken using the Leica TCS SP8 confocal microscope. The red and green fluorescence signals indicating the mRFP- and EGFP-expressing mitochondria, respectively, are seen. The mRFP (red channel) and EGFP (green channel) images were merged to observe mitochondria displaying only the red fluorescence. The images are representative of the three independent experiments whose data are plotted as bar graph showing the number of red mitochondria per cell. (B) Huh7 cells were cotransfected with the mitophagy reporter mito-mRFP-EGFP and JEV-NS4A-HA or empty-HA plasmids. The cells were fixed 48 h later, immunostained with JEV-NS4A antibody, and visualized under a Leica TCS SP8 confocal microscope. The red and green fluorescence signals indicating the mRFP- and EGFP-expressing mitochondria, respectively, are seen. The red (mRFP) and green (EGFP) channels were merged to detect mitochondria exhibiting only red fluorescence indicative of complete mitophagy. The images are representative of the three independent experiments whose data are plotted as bar graphs showing the number of red mitochondria per cell. Statistical analysis was performed using the Student’s *t* test (*, *P* ≤ 0.05; **, *P* ≤ 0.01; ***, *P* ≤ 0.001; ns, not significant).

Quantifying mitophagy flux is a reliable way of studying mitophagy. Completion of mitophagy involves the fusion of mitophagosomes with lysosomes resulting in the delivery of damaged mitochondria to the lysosomes for subsequent degradation and turnover (19). We used the mitophagy reporter mito-monomeric red fluorescent protein (mRFP)-enhanced green fluorescent protein (EGFP) to study the mitophagy flux in the JEV-infected cells. This reporter constitutes a tandem-tagged mRFP-EGFP protein fused to the mitochondrial localization sequence and assesses mitophagy based on the differential stabilities of mRFP and GFP in the acidic environment of the lysosomes. When mitochondria are delivered to the lysosomes, the GFP signal is rapidly quenched in the acidic environment, while the mRFP signal remains relatively stable for an extended time. Thus, the mitochondria harboring the reporter display only red fluorescence when delivered to the lysosomes, indicating complete mitophagy. In contrast, the mitochondria displaying both green and red fluorescence (yellow, upon the merger of both channels) indicate healthy mitochondria localized in the cytoplasm (20). To study the mitophagy flux, Huh7 cells were transfected with the mitophagy reporter, and 24 h posttransfection, the cells were infected with JEV for 48 h. As shown in Fig. 9A, the mock-infected Huh7 cells displayed mostly yellow mitochondria (see the green-red merged channel), suggesting a negligible level of mitophagy flux. However, the JEV-infected Huh7 cells showed a significant number of mitochondria with only red fluorescence (see the green-red merged channel), suggesting an enhanced level of mitophagy flux upon JEV infection. Huh7 cells treated with the mitochondrial decoupler CCCP to induce mitophagy also displayed a significantly high number of mitochondria with only red fluorescence (Fig. 9A). Quantification of mitophagy flux by determining the fraction of mitochondria displaying only the red fluorescence (mitochondria delivered to the lysosomes for degradation) clearly showed an enhanced mitophagy level in JEV-infected cells in comparison to that in the mock-infected cells (Fig. 9A, right panel).

Next, we examined if JEV-NS4A alone was sufficient to induce mitophagy. For this, Huh7 cells were cotransfected with the mitophagy reporter mito-mRFP-EGFP and JEV NS4A-HA expression vector or the empty vector (empty-HA). The JEV-NS4A-transfected cells showed a fraction of mitochondria with only red fluorescence significantly higher than that of the cells transfected with the empty vector (Fig. 9B). Together, these data indicate that JEV infection of mammalian cells induces mitophagy and JEV-NS4A alone is capable of inducing mitophagy.

### Mitochondrial fission and mitophagy are important for JEV propagation.

**FIG 10:**
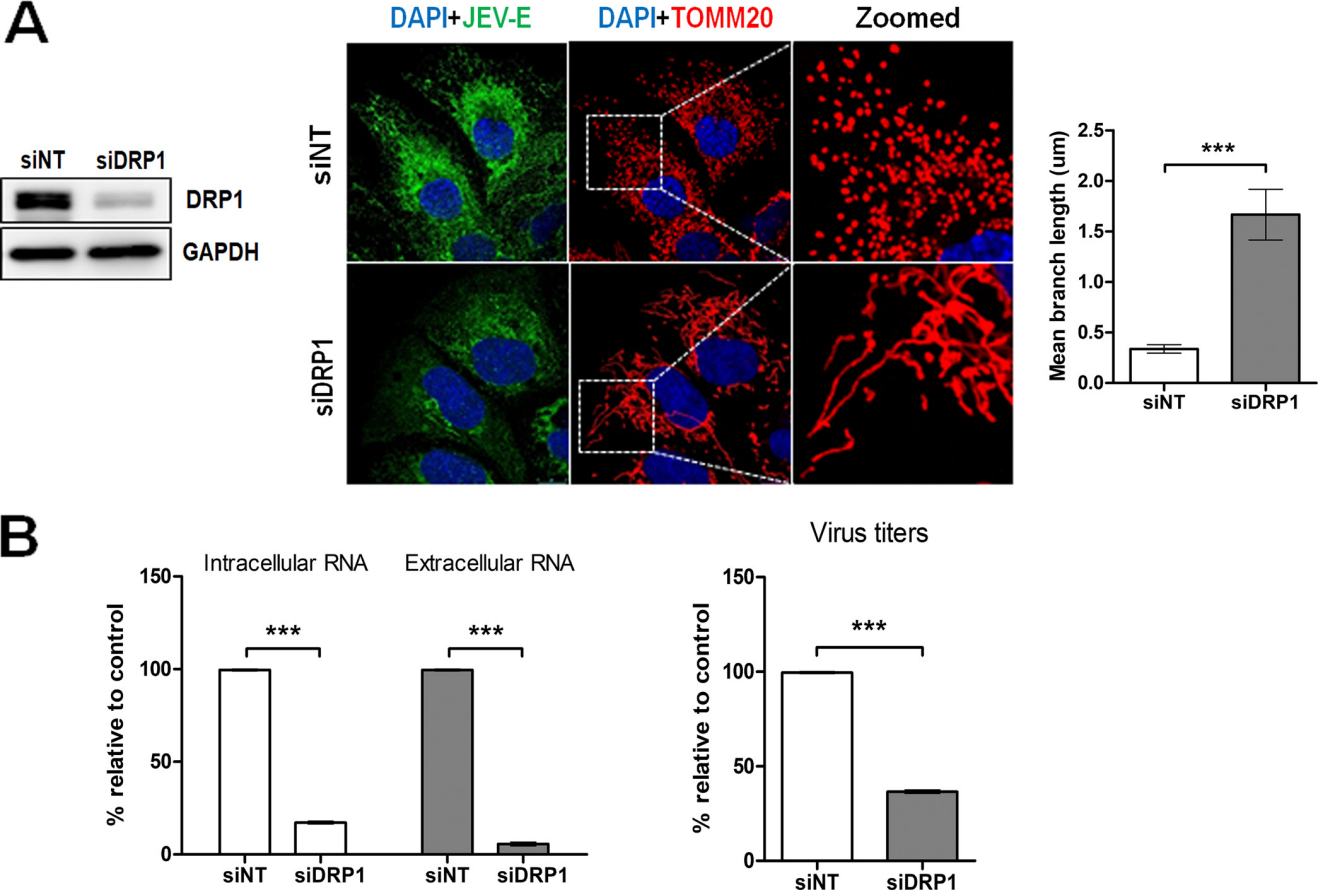
Role of mitochondrial fission in JEV life cycle. (A) Huh7 cells were treated with indicated siRNA, and 48 h later, the levels of DRP1 were determined by Western blotting. The siRNA-treated cells were infected with JEV (MOI 3) and 48 h later fixed with 4% PFA. Cells were immunostained for TOMM20 and JEV-E proteins. Images taken using the Leica TCS SP8 confocal microscope are shown. The mean mitochondrial branch length in siNT- and siDRP1-treated cells infected with JEV was quantified by Image J. The images are representative of the three independent experiments whose data are plotted as bar graphs. (B) The siRNA-transfected cells were infected with JEV (MOI 3) at 48 hpi, and 48 h later, the culture supernatant and cells were harvested. The total RNA isolated from the cells and culture supernatants were subjected to qRT-PCR to estimate the intracellular and extracellular JEV genome copy numbers. The virus titers in the culture supernatants were determined by the FFU assay. Statistical analysis was performed using the Student’s *t* test (*, *P* ≤ 0.05; **, *P* ≤ 0.01; ***, *P* ≤ 0.001; ns, not significant).

Our observations suggest that JEV promoted Drp1 activation and mitochondrial fragmentation. To further characterize the role of Drp1 in JEV life cycle, we performed a knockdown experiment. Small interfering RNA (siRNA)-mediated Drp1 silencing resulted in nearly 80% knockdown in Drp1 expression (Fig. 10A). Drp1 silencing resulted in significant inhibition of mitochondrial fragmentation in JEV-infected cells (Fig. 10A). This suggests that JEV infection-associated mitochondrial fragmentation is mediated by Drp1. To determine the significance of mitochondrial fragmentation in the JEV life cycle, DRP1 knockdown Huh7 cells were infected with JEV for 48 h. The levels of viral RNA in the cells, culture supernatant, and extracellular virus titers were estimated. Drp1 knockdown led to a significant reduction in both the intracellular and extracellular viral genome copy numbers (Fig. 10B). Drp1 knockdown also led to more than 50% inhibition in infectious viral titers (Fig. 10B).

**FIG 11:**
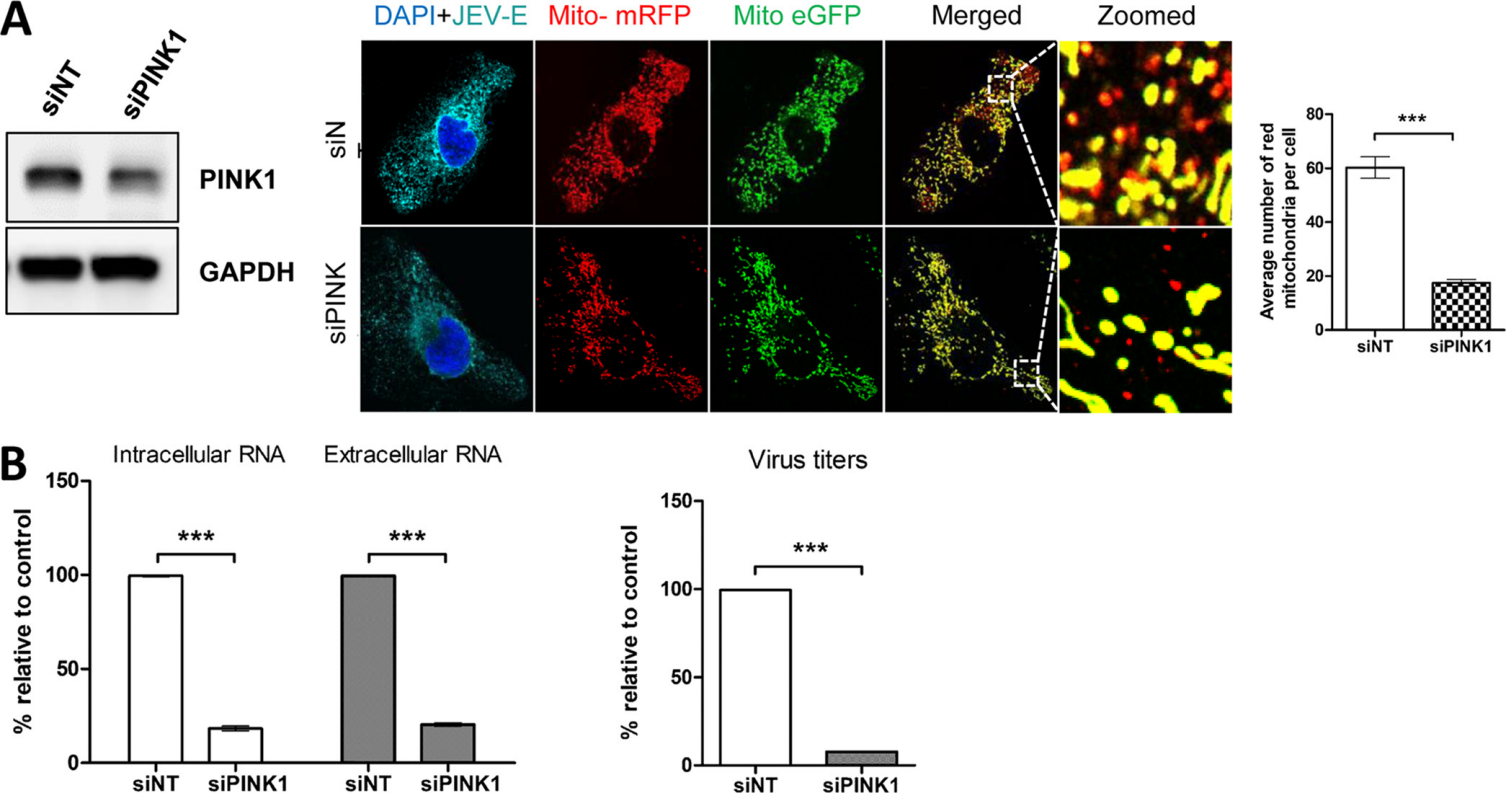
Role of mitophagy in JEV life cycle: (A) Huh7 cells were treated with indicated siRNA, and 48 h later, the levels of PINK1 were determined by Western blotting. The siRNA-transfected cells were transfected 16 h later with the mitophagy reporter plasmid mito-mRFP-EGFP and infected with JEV (MOI 3) 16 h later. The cells were fixed 36 h later with 4% PFA and immunostained for JEV-E protein. Images taken using the Leica TCS SP8 confocal microscope are shown. The number of mitochondria displaying only the mRFP fluorescence was quantified by Image J. The images are representative of the three independent experiments whose data are plotted as bar graphs showing the number of red mitochondria per cell. (B) Forty-eight hours posttransfection, the siRNA-transfected cells were infected with JEV (MOI 3), and 48 h later, the culture supernatant and cells were harvested. The total RNA isolated from the cells and culture supernatants were subjected to qRT-PCR to estimate intracellular and extracellular JEV genome copy numbers. The virus titers in the culture supernatants were determined by the FFU assay. Statistical analysis was performed using the Student’s *t* test (*, *P* ≤ 0.05; **, *P* ≤ 0.01; ***, *P* ≤ 0.001; ns, not significant).

We have observed that JEV infection led to PINK1 and Parkin activation and enhanced levels of mitophagy. To decipher the impact of PINK1 knockdown on JEV life cycle, we silenced PINK1 expression. siRNA-mediated silencing of PINK1 resulted in nearly 50% knockdown efficiency (Fig. 11A). Upon JEV infection, the PINK1 knockdown Huh7 cells displayed levels of mitophagy flux significantly lower than those of the JEV-infected Huh7 transfected with nontargeting control siRNA (Fig. 11A). Analysis of JEV genome copy and infectious viral titers suggested that PINK1 knockdown negatively affected JEV replication, resulting in nearly 80% inhibition in the intracellular and extracellular JEV genome levels and similar reduction in infectious virus titers (Fig. 11B). Overall, these data demonstrate the proviral influence of mitochondrial fission and mitophagy in JEV dissemination in Huh7 cells.

## DISCUSSION

Protein-protein interaction networks are an important ingredient for the system-level understanding of cellular processes. Mapping of the viral and host cell protein interactions has been used to provide insights into the viral pathogenesis and identify novel antiviral targets. We used the Y2H system for identifying host proteins that might interact with JEV-NS4A protein, as it can detect even weak and transient protein interactions *in vivo*. In our Y2H screen, 75 mouse proteins showed up as the potential interacting partners of JEV-NS4A. The STRING database, which imports data from experimentally derived protein-protein interactions and computationally predicted interactions, was used for the NS4A interactome mapping (21). The GO enrichment of our JEV-NS4A interactors showed significant enrichment of the mitochondrial component (GO: 0005739), highlighting the role of mitochondria in the JEV life cycle.

Many studies have shown that the flavivirus NS4A is majorly involved in membrane rearrangement and formation of the viral replication complex juxtaposed to the endoplasmic reticulum (ER) membranes (22, 23). The HCV-NS4A protein has been shown to localize on the mitochondria and surrounding ER cisternae, whereas dengue-NS4B has been shown to associate with mitochondrial proteins and modulate mitochondrial dynamics (24–26).

Mitochondrial dynamics has been shown to play an integral role in the regulation of mammalian cell homeostasis, metabolism, and innate immune signaling and has been implicated as a significant player in driving the outcome of several viral infections, including hepatitis B, hepatitis C, and dengue (24, 27). A recent study reported that dengue virus-infected insect cells modulated mitochondrial physiology to establish persistent infection (28). Abnormal mitochondrial dynamics is a common factor associated with mitochondrial dysfunction and neurodegeneration (29). Our GO-enrichment analysis revealed the identified host interactors to be significantly enriched in the Parkinson’s and Alzheimer’s disease pathways (KEGG pathways). In this context, it is important to note that JE disease has been associated with Parkinson’s-like neuropsychiatric sequelae (30). Furthermore, mitochondrial dysfunction has been strongly implicated in the etiology of Parkinson’s disease (18). Therefore, we decided to look into the role of the five genes enriched for the Parkinson’s pathway. Interestingly, further analysis revealed that all proteins were of mitochondrial membrane origin. Of them, PINK1 has been implicated in performing a neuroprotective role against a wide range of *in vitro* and *in vivo* insults (31–36). Patients with homozygous or compound heterozygous mutations in PINK1 exhibit neurodegeneration as the primary manifestation of their diseased state (37). Hence, we speculated that PINK1 might be relevant to the neuropathology associated with JE patients. This led us to investigate the significance of JEV-NS4A interaction with PINK1. The *in silico* studies provided a strong indication of the JEV-NS4A interaction with PINK1 where a stable adduct between PINK1 and NS4A with high docking energy (−75.0 kcal/mol) was obtained. Further, we provided experimental evidence of JEV-NS4A interaction with PINK1 in JEV-infected cells through confocal microscopy and coimmunoprecipitation methods. Additionally, we used the proximity ligation assay that detects the protein-protein interactions *in situ* at distances of <40 nm with high sensitivity and specificity (38, 39). Altogether, these data provided robust evidence for JEV-NS4A interaction with PINK1 during the virus infection.

Mammalian PINK1 is a 581-residue protein, with an N-terminal mitochondrial targeting sequence, a transmembrane helix, a serine/threonine kinase domain, and a C-terminal regulatory domain well known for its role in mitophagy (40). Under the normal steady-state conditions, the full-length ~63- kDa PINK1 is imported through the translocases of the outer and the inner mitochondrial membranes and cleaved sequentially, generating a ~52-kDa form (16, 41). This N-terminal-deleted PINK1 is released into the cytosol and is subsequently degraded by the ubiquitin-proteasome system (42, 43). This continuous import and degradation cycle yields very low to undetectable levels of full-length PINK1 in healthy cells. In this study, we found that JEV infection leads to a decrease in the expression of PINK1 protein despite the severalfold increase in the PINK1 transcript levels. Further experiments revealed that JEV-NS4A alone was sufficient to reduce the PINK1 expression levels. During mitochondrial injury, a decrease in the cleaved form is associated with a concomitant increase in the full-length PINK1. However, in JEV infection we did not observe accumulation of the full-length protein, suggesting that the PINK1 levels are regulated at the posttranscriptional stages. In correlation, we did observe PINK1 accumulation upon bafilomycin treatment of JEV-infected cells (see Fig. S2 in the supplemental material).

Depletion of the cleaved PINK1 is also important as recent studies implicate the role of the cleaved PINK1 fragments in determining neuronal cell homeostasis (44). The protein is neuroprotective (31) and plays a role in regulating cytosolic signaling cascades that promote cell survival (45). It also helps in dendritic arborization and neuron differentiation/neurite maintenance, through cooperation with valosin-containing protein (VCP) and protein kinase A (PKA) signaling pathway (46, 47). Synaptic dysfunction and dendritic pathology contribute to the early stages of neurodegeneration (48), suggesting that reduction in the levels of the cleaved PINK1 during the JEV infection may contribute to the development of Parkinson’s disease-like manifestations in the JE patients (30).

Our study showed that the JEV infection of the Huh7 cells perturbed mitochondrial dynamics with an overall decline in the mitochondrial branch length and footprint. The morphology of the mitochondrial network is a result of a delicate balance between mitochondrial fission and fusion events. Several studies show that PINK1 may alter this fission-fusion balance, and the PINK1 deficiency has been shown to favor mitochondrial fission, although a few contradictory observations are also reported (49–51). In our experiments, we noted the downregulation of PINK1 protein following the virus infection. It can be speculated that the reduced levels of PINK1 during JEV infection lead to mitochondrial fission contributing to neuropathology (52). In support of this, we observed that blocking JEV-mediated mitochondrial fragmentation resulted in a significant decline in JEV propagation. Previous studies have shown that the tubular mitochondrial network elicits a stronger antiviral response in contrast to the fragmented mitochondria (53, 54). We speculate that the DRP1 silencing in the JEV-infected cells led to a stronger antiviral response, thereby reducing replication of the virus. This is consistent with the published report showing an enhanced antiviral signaling and reduction in the HCV titers in the DRP1-silenced HCV-infected hepatic cells (55).

Many viruses induce mitophagy as a prosurvival process to avoid imminent cell death through apoptosis and to downregulate mitochondrial antiviral signaling (54, 56). In this study, we observed that the JEV infection of the cell triggered mitophagy. However, we observed a decrease in the PINK1 protein levels in the JEV-infected cells, which may be a consequence of an overall decrease in the mitochondrial mass due to the enhanced levels of mitophagy during JEV infection. This notion is supported by the fact that the bafilomycin treatment of the JEV-infected cells led to the accumulation in the levels of both PINK1 and PARKIN, suggesting that the JEV-induced mitophagy may be mediated through the PINK1-PARKIN axis. Recent studies have also revealed several functions of PINK1 independent of its role in mitophagy (31, 45–48). We observed that the PINK1 knockdown led to the suppression of JEV replication, whereas overexpression of PINK1 promoted JEV replication. This suggested that PINK1 may have a proviral role during JEV infection which may be mediated through its role in PINK1-PARKIN-dependent mitophagy or mitophagy-independent functions of PINK1. Further experiments are required to establish the PINK1-dependent nature of mitophagy, as these pathways may be cell-type dependent (57). Complete mitophagy has previously been reported during infections with HBV, HCV, influenza A, and Newcastle disease viruses (53, 58). In this study, we also observed a complete mitophagy in the JEV-infected Huh7 cells using a mitophagy flux reporter. Further, we observed that JEV-NS4A alone could induce mitophagy.

To our knowledge, this is the first evidence of JEV-NS4A affecting the mitochondrial dynamics through its interaction with PINK1, the protein that is involved primarily in mitochondrial quality control but has been recently associated with many novel functions, including the regulation of antiviral immune response (59). Several neurotropic viruses have been associated with both acute and chronic Parkinsonism (60). The JEV-NS4A and PINK1 interaction may be playing an important role in JE-associated neuropathology. However, further studies are needed to elucidate the precise effect of this interaction on the JE disease manifestation. The knowledge obtained may prove useful in understanding the aspect of JE neuropathology and facilitate the development of novel therapeutics.

## MATERIALS AND METHODS

### Molecular cloning for the Y2H assay.

The cDNA for the full-length JEV-NS4A was amplified by PCR using gene-specific primers flanked by *Sfi* I linkers on both ends (forward primer: attaacaaggccattacggcctcagccaagcttcatagaggtgctc; reverse primer: aactgattggccgaggcggcccgctgccactccaactacggtc) and cloned into the pGEM-T vector using the TA cloning method. The JEV-NS4A cDNA was then transferred to a Lex A-VP16 DNA-binding domain-based vector pBT3N using the *Sfi* I restriction sites.

### Yeast transformation.

Saccharomyces cerevisiae strain NMY51: MATa his3200 trp1-901 leu-2-3,112 ade2 LYS2::(lexAop)4-HIS3 ura3::(lexAop)8-*lacZ* ade2::(lexAop)8-ADE2 GAL4 was used (61) in all the experiments. Yeast cells were grown using the standard microbial techniques and media (62). The media used were yeast extract-peptone-dextrose plus adenine medium (YPAD), synthetic defined dropout medium (SD), and various minimal dropout media designated by the amino acid that is lacking (e.g., −leu −trp −his −ade media lack the respective amino acids). The lithium acetate (LiOAc)-mediated transformation was done to transfer the JEV-NS4A plasmid into the NMY51 strain of S. cerevisiae (63).

### The Y2H screen.

Large-scale yeast library transformation was performed as described by the manufacturer (DUALmembrane starter kit, Dualsystems Biotech). For the functional assay, the bait was used to cotransform the yeast with a positive-control vector pOst1-Nubl and an empty library vector pPR3-N. The transformed yeast colonies were obtained with the positive control, but none were obtained with the negative control in the SD medium lacking leucine, tryptophan, histidine, and adenine (SD-LWHA) plates, thus ruling out auto-activation. For the pilot screen, yeast cotransformed with the bait construct and the empty prey library vector pPR3-N was titrated with 3-aminotriazole (3-AT), a competitive inhibitor of the HIS3 gene product, to provide more stringent conditions, but no colonies could be seen even in the absence of 3-AT for this particular bait.

For the library screen, the starter culture of the bait-construct-containing yeast, grown in the SD-LE medium at 30°C by overnight shaking, was used to seed 50 mL of the secondary culture to reach the final optical density at 546 nm (OD_546_) of 0.6. The cells were then pelleted, washed in 1× Tris-EDTA (TE), repelleted, and resuspended in 1 mL LiOAc/TE master mix (100 mM LiOAc, 0.5× TE [pH 8.0]) and incubated at room temperature for 10 min to generate the competent bait-containing yeast cells. These nascent competent cells were transformed using 10 μg Mus musculus brain cDNA prey library (DUALmembrane starter kit, Dualsystems Biotech) and 100 μg salmon sperm DNA. Following this, 700 μL polyethylene glycol (PEG)/LiOAc mix (100 mM lithium acetate, 40% PEG 3350, 1× TE) was added, vortexed, and incubated at 30°C for 30 min. After that, 88 μL dimethyl sulfoxide (DMSO) was added and mixed well, followed by a 7- min heat shock at 42°C. The cells were then centrifuged at 1,500 × *g* for 10 min, resuspended in 5 mL 2× YPAD medium, allowed to recover at 30°C for 2 h with slow shaking (150 rpm), again centrifuged, washed, and resuspended in 2.1 mL TE. The recovered cells were plated on 20 90-mm SD-LWHA plates and 3 SD-LW plates (to confirm the existence of transformants) and incubated at 30°C. After 4 days, the SD-LW plates with the transformants were removed and stored at 4°C, whereas the SD-LWHA plates were allowed to grow for 10 days. To verify the putative interactions between the bait protein and the prey protein from the cDNA library, positive clones from the screen were restreaked on the SD-LWHA plates. After growth for 5 days, the prey plasmids were recovered by Zymolyase treatment (64).

### β-Galactosidase activity assay.

The qualitative β-galactosidase activity assays were carried out using the colony-lift filter method. A nitrocellulose filter was placed on the colonies for 10 sec, dipped in liquid nitrogen for 1 min, and then thawed at room temperature. It was placed on a presoaked Whatman (1 mL Z-buffer and 17.5 μL X-Gal solution in dimethylformamide [DMF] at 20 mg/mL) and incubated at 30°C for 10 to 60 min. The appearance of the blue color indicated the positive colonies. Finally, the identity of the positive clones was determined by sequencing and using the Basic Local Alignment Search Tool (BLAST).

### Interactome mapping.

The interactome mapping was done using the STRING (Search Tool for the Retrieval of Interacting Genes/Proteins) tool (version 10.5), which is a biological database and web resource of known and predicted protein-protein interactions. The gene enrichment analysis was also done using the STRING tool.

### Computational methods.

In the absence of the crystal structure of JEV-NS4A, template was taken from I-TASSER. Although the crystal structure of human PINK1 is reported (PDB-ID 6EQI), some of its key regions, like a helix, hinge, and linkers, were not crystalized. Therefore, the complete hPINK1 protein structure was generated by homology modeling. The robustness of each model was evaluated through ERRAT, PROSA, and Ramachandran plot, and molecular dynamics simulations (100 ns) were done to allow conformational relaxation of the protein structures. The most stable state of proteins was picked (lowest close to RMSD) to perform the protein-protein docking studies through three different algorithms (PyDock, Swarmdock, Cluspro). Clustering of the docked poses was conducted to generate the most likely complex of PINK1-NS4A based on several conformers and the lowest binding energy (−75 to −80 kcal/mol).

### Antibodies.

The following primary antibodies were used: rabbit anti-JEV-NS4A (GeneTex, GTX132028), rabbit anti-PINK1 (Abcam, ab23707), mouse anti-PINK1 (Abcam, ab75487, ab186303), rabbit anti-TOMM20 (Santa Cruz, sc-11415), mouse anti-COX2 (Santa Cruz, sc-514489), rabbit anti-COX4 (CST, no. 4844), rabbit anti-mitofusin-1 (CST, no. 14739), rabbit anti-mitofusin-2 (CST, no. 11925), rabbit anti-DRP1 (CST, no. 8570), rabbit anti-p-DRP1 (Ser 616; CST, no. 4494), rabbit anti-p-PARKIN (S65; CST, no. 36866S), mouse anti-PARKIN (CST, no. 4211S), rabbit anti-FACL4 (ab155282), rabbit anti-OPA1 (CST, no. 67589), goat anti-SERCA2 (Abcam, ab183531), rabbit anti-β-actin (CST, no. 4970), rabbit anti-HA (GeneTex, GTX115044), rabbit anti-GAPDH (GeneTex, GTX100118), rabbit anti-GFP (Cloud-clone Corp., PAD025Ge07), and GFP-trap antibody (Chromotek, GTA-20 GFP-TRAP). The horseradish peroxidase (HRP)-conjugated secondary antibodies used were donkey anti-mouse and anti-rabbit antibodies (Jackson Immunochemicals, 711-035-152 and 715-035-150). The secondary antibodies used in immunofluorescence experiments were chicken anti-mouse antibody-Alexa fluor 488 and goat anti-rabbit antibody-Alexa fluor 568 (NextGen Life Sciences, A21200 and A11011).

### Plasmids.

For expressing the HA-tagged JEV-NS4A, the JEV-NS4A cDNA was cloned in the pSELECT-CHA-zeo vector containing the HA tag at its C terminus. The plasmid pcDNA-DEST53 PINK1 N-GFP (Addgene, 13315, deposited by Mark Cookson) was used for expressing the GFP-tagged PINK1, and plasmid pAT016 (p-mito-mRFP-EGFP) was a kind gift from Andreas Till (20).

### Virus.

The P20778 strain of JEV was used in all experiments. The C6/36 cell monolayers at 70 to 80% confluence were infected with JEV at a multiplicity of infection (MOI) of 0.01. After 1 h, cells were washed with phosphate-buffered saline (PBS) and incubated in the L-15 medium with 2% fetal bovine serum (FBS). Culture supernatant was collected at 60 h postinfection (hpi) and titrated by plaque formation on porcine stable kidney (PS) monolayers (65) or foci-forming unit assay (see below).

### Mice experiments.

Three-week-old BALB/c mice (*n* = 4) were mock-infected or infected with 100 μL of 10^8^ foci-forming units (FFU)/mL of JEV injected intraperitoneally. Mice were sacrificed 5 days postinfection when JEV-specific symptoms emerged. Liver tissue isolated from the mice was homogenized to prepare the lysate and used for Western blotting. The animal experiment had the approval of the Institutional Animal Ethical Committee from ILS, Bhubaneswar (no. ILS/IAEC-153-AH/FEB-19).

### Cell culture and plasmid transfection.

Huh7 and HEK293T cells were grown in Dulbecco modified Eagle medium (DMEM). Aedes albopictus-derived C6/36 cells were grown in the L-15 medium. The porcine kidney (PS) cells were grown in minimal essential medium (MEM) supplemented with 2 mM l-glutamine, 100 μg/mL penicillin/streptomycin, and 10% heat-inactivated fetal bovine serum (FBS) at 37°C in 95% humidified air and 5% CO_2_. The cells were transfected with 1 μg plasmid DNA using lipofectamine 2000 (Invitrogen, 11668-027) and incubated for 48 h in an antibiotic-free medium before processing.

### siRNA transfection.

Small interfering RNA (siRNA) pools used in this study were siGENOME SMARTpool for PINK1 (M-004030-020005), Accell Human DNM1L (10059) for DRP1 (E-012092-00-0005), and ON-TARGETplus nontargeting pool as the control (Dharmacon, D-001810-10-05). The cells were transfected with siRNA (30 nM) for the indicated times using the DharmaFECT1 transfection reagent (Dharmacon, T-2001-01) according to the manufacturer’s instructions.

### FFU assay.

Vero cells were seeded at a density of ~10,000 cells/well in a 96-well plate 24 h before infection. For the titer assays, 10-fold serial dilutions of the culture supernatants containing the virus were prepared and used to infect the cell monolayers at 37°C. After 2 h, the inoculum was aspirated, and cells were washed with PBS and incubated for a further 48 h. We detected foci by immunostaining the fixed cells by antibody against JEV-prM (GeneTex, Irvine, CA, USA) at 1:200 dilution or 1 μg/mL, followed by three washes with PBS and staining with respective Alexa fluor conjugated secondary antibody (Thermo Fisher, Waltham, MA, USA) for 1 h at room temperature. The foci were visualized using an Olympus DX58 fluorescence microscope, and titers were estimated as FFU/mL of the culture supernatant.

### qRT-PCR.

For determining the JEV genome copies, total RNA was isolated from the JEV-infected or mock-infected cells or preclarified culture supernatants using the TRIzol reagent (Life Technologies, Carlsbad, CA, USA) as per the manufacturer’s instructions. Viral genome copies were measured by quantitative real-time (qRT-PCR) using a one-step RT-PCR kit (TaKaRa Scientific, Japan) in Quant Studio 6 real-time PCR system following the manufacturer’s protocol. The JEV forward primer 5′-ggtgtaaggactagaggttagagg-3′ and reverse primer 5′-ggtgtaaggactagaggttagagg-3′ along with the JEV probe (5′-Fam-cccgtggaaacaacatcatgcggc-TAMRA-3′) were used. The absolute quantification of viral genome copies was assessed using the standard curve prepared using the known quantities of a plasmid harboring the cDNA coding for the JEV genome segment targeted by the primer-probe sets.

### Immunostaining, fluorescence microscopy, and image processing.

For protein localization studies on mitochondria, cells were pulsed with 200 nM MitoTracker Red (Invitrogen, M7513) for 1 h before fixing in 4% paraformaldehyde (PFA) and permeabilizing using 0.3% Tween 20 for 20 min at room temperature. Blocking was done with 2 mg/mL bovine serum albumin (BSA; Sigma, A7906) in PBS for 1 h before overnight incubation with primary antibodies, followed by incubation with Alexa-coupled 488/568 secondary antibodies. Coverslips were mounted using Fluoroshield with DAPI (4′,6-diamidino-2-phenylindole; Genetex, GTX30920). Other immunofluorescence experiments were also done similarly, except for treatment with MitoTracker Red. For the colocalization studies, Z-stacks were acquired at 0.25 μm per slice by sequential scanning with a 60× lens objective on a confocal microscope. FluoView and LasX software were used to generate cross-sectional and maximum intensity projection images to calculate Pearson’s coefficient of colocalization between the two fluorophores. Colocalization values are represented as mean from 10 to 15 cells each from three independent experiments. Quantitative analysis of the mitochondrial length and mass was performed using the extension MINA in FIJI software. The mitochondrial length and footprint values are the averages derived from 15 and 7 cells, respectively. The colocalized spots were generated using the colocalization threshold macro in the Fiji software.

### Mitophagy flux assay.

To determine the mitophagy flux, we transfected Huh7 cells with the mitophagy reporter mito-mRFP-EGFP and infected them with JEV (MOI 1) at 24 h posttransfection. The cells were fixed at 48 hpi with 4% PFA for 15 min, followed by five washes with PBS. The cells were blocked and permeabilized with 3% BSA and 0.1% Triton X-100 in PBS for 1 h followed by overnight incubation with JEV-E specific primary antibody at 4°C. After three washes with PBS, cells were stained with Alexa fluor 647 conjugated secondary antibody. Cells were washed three times with PBS, and the coverslips were mounted onto the glass slides using Prolong Diamond anti-fade (Invitrogen). Images were taken using a Leica TCS SP8 confocal microscope. Quantitative analysis of the mitophagy flux was done manually by counting the red and yellow mitochondria in about 15 cells each for every condition in a typical experiment from three independent experiments. Percentage of red mitochondria with respect to total mitochondria in mock-infected, JEV-infected, and CCCP-treated (Sigma no. C2759, 20 μM, for 14 h, prefixation) Huh7 cells was calculated and plotted using GraphPad Prism software. For the NS4A mitophagy flux experiment, Huh7 cells were cotransfected with NS4A-HA or empty-HA plasmid along with the mitophagy reporter plasmid.

### Subcellular fractionation.

Mitochondria from mock-infected and JEV-infected Huh7 cells were isolated using the mitochondria isolation kit (Thermofisher Scientific, catalog no. 89874) as per the manufacturer’s instructions. Briefly, 2 × 10^7^ cells were resuspended in isotonic buffer and the cell suspension was transferred to the Dounce homogenizer and subjected to 50 to 60 strokes to promote cell lysis/rupture. The homogenized cell suspension was subjected to differential centrifugation to obtain the crude mitochondrial pellet and postmitochondrial supernatant (PMS). The PMS was subjected to centrifugation at 40,000 × *g* for 2 h at 4°C to obtain the microsomal pellet and the cytosolic fraction as the supernatant. The entire procedure was carried out on ice, and the pellets obtained were immediately resuspended in RIPA buffer to ensure complete solubilization.

### PLA.

The Huh7 cells seeded on sterile coverslips were mock-infected or infected with JEV (MOI 5). The cells were fixed at 24 h pi, permeabilized, and blocked, as explained above. The coverslips were incubated in the primary antibodies (JEV-NS4A and PINK1) overnight and subsequently treated according to the manufacturer’s protocol (Duolink *in situ* PLA kit, Sigma-Aldrich, DUO92101-1KT). The corresponding negative controls of the virus-infected or transfected cells were subjected to overnight incubation with JEV-NS4A antibody only. The secondary antibody (anti-rabbit PLUS and anti-mouse MINUS) PLA probes were incubated at 1:5 dilution for 1 h at 37°C, followed by incubation in the ligation solution for 30 min at 37°C. Rolling circle amplification was done by incubating in the amplification-polymerase solution for 100 min at 37°C, and the coverslips were mounted using the Duolink *in situ* mounting medium with DAPI. The imaging was done as explained above, and the quantitation was done by spot counting the number of red signals per cell using ImageJ software.

### Western blotting.

Mock-infected, JEV-infected, or CCCP-treated (20 μM for 14 h) cells were washed in PBS and lysed in RIPA buffer (50 mM Tris-HCl [pH 8], 150 mM NaCl, 1% TritonX-100, 0.25% sodium deoxycholate, and 0.1% SDS) containing protease and phosphatase inhibitor cocktail (Thermo Scientific). Protein estimation was done using the bicinchoninic acid (BCA) protein assay kit (TaKaRa, T9300A). Equal amounts of protein extracts were separated on 10% polyacrylamide gels and transferred to nitrocellulose membranes (MDI, SCNJ8102XXXX101) for Western blotting. The membranes were blocked using 5% skimmed milk for 1 h at room temperature before incubation with primary antibodies at 4°C overnight. After three washes of 10 min each with PBS containing 0.1% Tween 20, the blot was incubated for 1 h at room temperature with respective HRP-conjugated secondary antibody. For detection of the protein bands, the enhanced chemiluminescent substrate (Bio-Rad, catalog no. 1705060) was added for 5 min in the dark and then the blot was visualized on a Gel-doc system. The membrane was also probed with an anti-GAPDH antibody to check for the level of GAPDH, which was used to normalize the expression of other cellular proteins. To determine whether mitophagy is the cause of downregulation of mitochondrial proteins, mock-infected and JEV-infected Huh7 cells were treated with 125 nM bafilomycin A1 (Cell Signaling Technology, catalog no. 54645S) for 8 h after 30 h of JEV infection. Cells were lysed using RIPA lysis buffer, and the resulting lysates were subjected to Western blotting.

### Immunoprecipitation.

HEK293T cells were transfected with the plasmid expressing PINK1 fused to GFP. The whole-cell lysates were prepared 48 h later in lysis buffer containing 10 mM Tris/HCl (pH 7.5), 150 mM NaCl, 0.5 mM EDTA, 0.5% NP-40 supplemented with protease inhibitors, and 1 mM phenylmethylsulfonyl fluoride (PMSF) and left on ice for 30 min. The cell lysate was centrifuged at 20,000 × *g* for 10 min at 4°C, and the supernatant was incubated with GFP-Trap beads (ChromoTek, Planegg-Martinsried, Germany). The mix was tumbled for 1 h and centrifuged at 2,500 × *g*, and the supernatant was discarded. The beads were washed twice with PBS at 4°C and then resuspended and boiled at 95°C for 10 min in 100 μL 2× SDS-sample buffer. GFP-Trap beads were collected by centrifugation at 2,500 × *g*, and the supernatant was used for performing SDS-PAGE followed by Western blotting.

### Statistical analysis.

Statistical analysis was performed using the unpaired Student’s *t* test, and a *P* value of ≤0.05 was considered significant.
