# Supplementary material for: Japanese Encephalitis Virus NS4A Protein Interacts with PTEN-Induced Kinase 1 (PINK1) and Promotes Mitophagy in Infected Cells
Source: Microbiol Spectr. 2022 May 23;10(3):e00830-22. doi: 10.1128/spectrum.00830-22 (PMC9241661; doi:10.1128/spectrum.00830-22)
Supplement: SUPPLEMENTAL FILE 1 — Supplemental material. Download spectrum.00830-22-s001.pdf, PDF file, 3.9 MB [file spectrum.00830-22-s001.pdf]

Table S1. List of JEV NS4A interactors

Sl. No.	Gene symbol	NCBI GENE ID	Sl. No.	Gene symbol	NCBI GENE ID
1	ABCD3	5825	39	MRPL33	9553
2	AHCYL2	23382	40	MRPL48	51642
3	ARHGAP12	94134	41	MRPL49	740
4	ARFIP2	23647	42	MRPS14	63931
5	ANGEL2	90806	43	NDN	4692
6	ASB13	79754	44	NIPSNAP1	8508
7	ATP5B	506	45	OMG	4974
8	AUTS2	26053	46	NUPR1	26471
9	BSDC1	55108	47	PINK1	65018
10	CCDC49	54883	48	PJA2	9867
11	CCDC109B	55013	49	PLXNA2	5362
12	CCDC186	55088	50	PRDX3	10935
13	CPLX1	10815	51	PTPRN2	5799
14	COPG1	22820	52	RAG2	5897
15	COX6C	1345	53	RASGRP2	10235
16	CNTN1	1272	54	RBM8A	9939
17	CYP2E1	1571	55	RESP18	389075
18	DAZAP2	9802	56	RFWD3	55159
19	DLGAP1	9229	57	RGS5	8490
20	EIF3E	3646	58	RPL35A	6165
21	FAM32A	26017	59	RPS7	6201
22	FAR1	84188	60	SDHC	6391
23	FNIP1	96459	61	SLC7A3	84889
24	GHITM	27069	62	SPA17	53340
25	GLUL	2752	63	SELENOF	9403
26	GNL3L	54552	64	SSR4	6748
27	HAX1	10456	65	SSU72	29101
28	HIGD2A	192286	66	SUPT6H	6830
29	HSPA2	3306	67	TMEM30A	55754
30	ITM2A	9452	68	TMA7	51372
31	ITPR2	3709	69	TRAPPC11	60684
32	KCNAB2	8514	70	UHRF1BP1L	23074
33	LDOC1L	84247	71	UQCRC2	7385
34	LMO4	8543	72	VKORC1	79001
35	LZTS1	11178	73	YWHAQ	10971
36	MAP1B	4131	74	ZBTB41	360023
37	MAPKBP1	23005	75	ZMAT4	79698
38	MKRN1	23608			

Supplementary Figure S1

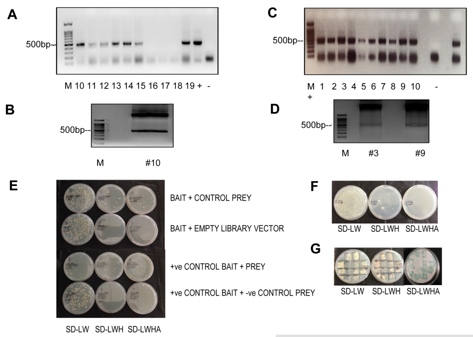

Supplementary Figure S2

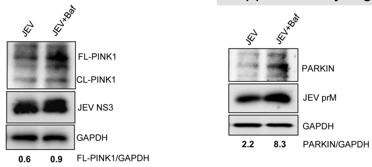

Supplementary Figure S3

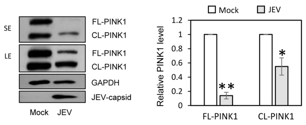

Figure Legends

Fig. S1: Cloning of JEV-NS4A for the Y2H screening. (A) The JEV-NS4A cDNA was RT-PCR amplified and cloned in the pGem-T vector. The Colony-PCR using the JEV-NS4A primers identified the putative clones. **(B)** Restriction digestion of plasmid DNA from colony #10 with *Sfi* I confirming an insert release of JEV-NS4A of 447 bp. **(C)** The JEV-NS4A cDNA released from the colony #10 DNA was cloned in plasmid pBT3-N (bait). The Colony-PCR using the JEV-NS4A primers identified the putative clone. **(D)** Restriction digestion with *Sfi* I of the plasmid DNA from colony #3 and #9 from the pBT3-N cloning confirmed the desired clones. **(E)** The bait plasmid was transferred to the competent NMY51 strain of yeast (deficient in leucine, tryptophan, histidine, and adenine pathways). The prey plasmid was then transferred to the bait-containing yeast (SD-LW) and checked for expression of the markers by selecting on histidine and adenine deficient plates. **(F)** Pilot screen to confirm that the bait clone was not self-activating. **(G)** Library screen showing up the interactors on selective plates and the positive X-Gal assay. The plasmid DNA from the interaction positive clones was transferred to E. coli strain DH5a to amplify and used for DNA sequencing.

Fig. S2. Mock- and JEV-infected (MOI 1) Huh7 cells at 30 h pi were treated with bafilomycin for an additional 8 h. The cell lysates were subjected to Western blot analysis for the mitochondrial proteins PINK1 (left panel) and PARKIN (right panel). GAPDH was used as the loading control and JEV prM and NS3 were used as the infection marker. The respective band intensities relative to GAPDH are shown below each blot.

Fig. S3. Three weeks old C57BL/6 mice (n=4) were JEV or mock-infected by the 10^7 PFU of JEV injected intraperitoneal. The brain tissues were harvested 5 days later and prepared as lysates. The left panels show the Western blot analysis of indicated proteins in JEV-infected mouse brain lysate**.** The JEV capsid was used as the infection marker and GAPDH was used as the internal loading control. The right panel shows quantification of protein band intensities of mouse tissue from the Western blots.
